# Migration of Kupffer's vesicle-derived cells is essential for tail morphogenesis in zebrafish embryos

**DOI:** 10.1242/dev.204791

**Published:** 2025-06-19

**Authors:** Jelmer Hoeksma, Jeroen den Hertog

**Affiliations:** ^1^ Hubrecht Institute – KNAW and University Medical Center Utrecht, Utrecht, The Netherlands; ^2^ Institute Biology Leiden, Leiden University, Leiden, The Netherlands

**Keywords:** Kupffer's vesicle, Geraniol, Tail morphogenesis, Zebrafish

## Abstract

A phenotypic screen of fungal filtrates on developing zebrafish embryos identified metabolites from the fungus *Ceratocystis populicola* to induce ectopic tail formation, including a split notochord and a duplicated caudal fin. Chemical analyses led to the identification of monoterpene alcohols, in particular geraniol, as the active compounds. Tüpfel long fin zebrafish embryos were more susceptible to geraniol-induced ectopic tail formation than Wild Indian Karyotpe zebrafish embryos. RNA-sequencing on tail buds of 15-somite-stage embryos revealed downregulation of essential genes of the retinoic acid signaling pathway. Differential expression of *cyp26a1, fgf8a* and downstream hox-genes was validated. Time-lapse imaging revealed that Kupffer's vesicle-derived cells failed to migrate after Kupffer's vesicle collapse upon geraniol treatment. These cells failed to merge with progenitors from the tail bud and contributed to an ectopic tail, expressing markers for presomitic mesoderm, somite and notochord tissue. Strikingly, ablation of Kupffer's vesicle by *tbxta*-morpholino injection rescued ectopic tail formation. Taken together, our data suggest that Kupffer's vesicle cells harbor tail progenitor capacity, and proper migration of these cells is essential for normal tail morphogenesis.

## Teaser

**Summary:** Inhibition of Kupffer's vesicle-derived cell migration affected tail morphogenesis and resulted in ectopic tail formation in zebrafish embryos.

## INTRODUCTION

Zebrafish (*Danio rerio*) is a versatile and powerful vertebrate model to investigate development and disease using molecular genetics and high throughput small molecule screens. Previously, we reported a phenotypic screen of 10,207 fungal filtrates using developing zebrafish embryos – this screen led to the identification of biologically active fungal compounds, including compounds which are routinely being used in the clinic (Hoeksma et al., 2019). In addition, we found that 25.2% of the active fungal filtrates induced tail defects. In a follow-up study, we focused on this category and identified the fungal compound cercosporamide as a bone morphogenetic protein (BMP)-signaling inhibitor, based on the dorsalized phenotype it induces in zebrafish tails (Hoeksma et al., 2020), demonstrating that fungal filtrates interfering with tail morphogenesis harbor compounds with interesting biological activities.

Many signaling pathways are activated during zebrafish tail morphogenesis, which together orchestrate proliferation, cell transitions and cell movements required to establish a fully functional structure. Crucial are the formation and tight regulation of morphogen gradients in all three dimensions. The BMP gradient is essential for dorsoventral patterning of the zebrafish tail (Row and Kimelman, 2009). BMP signaling inhibitors such as dorsomorphin and DMH-1 result in a dorsalized phenotype including loss of ventral fins, as does cercosporamide that we identified previously (Hao et al., 2010; Hoeksma et al., 2020; Yang and Thorpe, 2011; Yu et al., 2008). Similar dorsoventral patterning defects are observed in loss-of-function mutants of key genes of the BMP-signaling pathway, including *activin receptor-like kinase 2* (*alk2*; in zebrafish also known as *acvr1l*, *alk8* or *lost-a-fin*) (Bauer et al., 2001), its ligand, *bmp2* (Kishimoto et al., 1997) or intracellular factors such as *smad5* (Kramer et al., 2002). In contrast, overactivation of BMP-signaling results in a ventralized phenotype (Irmler et al., 2004).

The key gradient for left-right patterning in tail morphogenesis is the *nodal* (*ndr1*)/*spaw* gradient, which is initiated by Kupffer's vesicle, a fluid-filled transient structure located at the ventral posterior side of the embryo (Kupffer, 1868; Long et al., 2003). Cilia within Kupffer's vesicle generate an anti-clockwise fluid flow, which in turn induces asymmetric expression of signaling genes such as *spaw* and establish a *nodal*/*spaw* gradient (Essner et al., 2005). This is not only essential for left-right patterning of the tail, but also for asymmetrical organ placement elsewhere in the embryo. Impairment of this signaling pathway can be induced by various drugs (Levin, 2005) and causes tail defects, randomization of organ positioning and heart looping failure (Bonetti et al., 2014; Kramer-Zucker et al., 2005; Smith et al., 2011).

Finally, anterior-posterior patterning is controlled by antagonistic gradients of Wnt/Fgf and retinoic acid, which regulate each other through feedback mechanisms (Stulberg et al., 2012). Key genes in this process are *cyp26a1* (also known as *gir*) and *aldh1a2*. *Cyp26a1* is expressed in the presomitic mesoderm of the tail bud and degrades retinoic acid into retinoid metabolites (Chithalen et al., 2002; Kudoh et al., 2001; Roberts, 2020). *Aldh1a2* is expressed more anteriorly and produces retinoic acid (Pittlik and Begemann, 2012). High levels of retinoic acid inhibit Wnt/Fgf signaling and, conversely, expression of *aldh1a2* is inhibited by high levels of *fgf8a* (Draut et al., 2019). Furthermore, retinoic acid signaling regulates body axis extension and somitogenesis by controlling expression of downstream homeobox transcription factor genes, including hox and cdx family members (Shimizu et al., 2006). Fgf/Wnt signaling controls transcription factors such as *msgn1* and *tbx16*, which in turn regulate neuromesodermal stem cells that reside in the tail bud (Manning and Kimelman, 2015). Perturbations of retinoic acid signaling in zebrafish result in severe tail defects. For example, mutants of *cyp26a1* show a shortened and distorted tail (Emoto et al., 2005). Incubation of zebrafish embryos with retinoids causes severe pleiotropic defects including truncation and shortened body axis (D'Aniello et al., 2015; Minucci et al., 1996).

Taken together, morphogen gradients regulate signaling pathways in an intricate manner and shape the developing embryo. Modulating these signaling pathways may particularly affect tail formation. Small molecules that affect zebrafish tail morphogenesis may provide new insights into morphogenesis on the one hand and into signaling pathways on the other.

Here, we found ectopic tail formation in response to a fungal filtrate. We established that monoterpene alcohols, in particular geraniol, induced ectopic tail formation during a 4 h window at tail bud stage, most potently in Tüpfel long fin (TL) embryos. We show that the mode-of-action of geraniol is distinct from known ectopic-tail-inducing compounds such as BMP-pathway inhibitors and para-coumaric acid methyl ester (pCAME; Gebruers et al., 2013; Yang and Thorpe, 2011). Using transcriptomics of tail buds at tail bud stage, we found genes involved in retinoic acid signaling to be downregulated in geraniol-treated embryos. Yet, overexpression of these genes or co-incubation with enhancers of retinoic acid signaling did not rescue the phenotype. Next, time-lapse imaging revealed a migration defect of Kupffer's vesicle derived cells (KVDCs), suggesting that impaired migration of these cells underlies geraniol-induced ectopic tail formation. When looking at later stages, we found expression of presomitic mesoderm, somite and notochord markers in ectopic tails, but no neural markers. Finally, ablation of Kupffer's vesicle through *tbxta*-morpholino (MO) injection in mid-blastula-stage embryos blocked ectopic tail formation upon geraniol treatment. We conclude that former Kupffer's vesicle epithelial cells function as a distinct cluster of tail progenitors and their migration is essential for normal tail morphogenesis.

## RESULTS

### Monoterpenes cause ectopic tail formation in zebrafish embryos

**Fig. 1.**
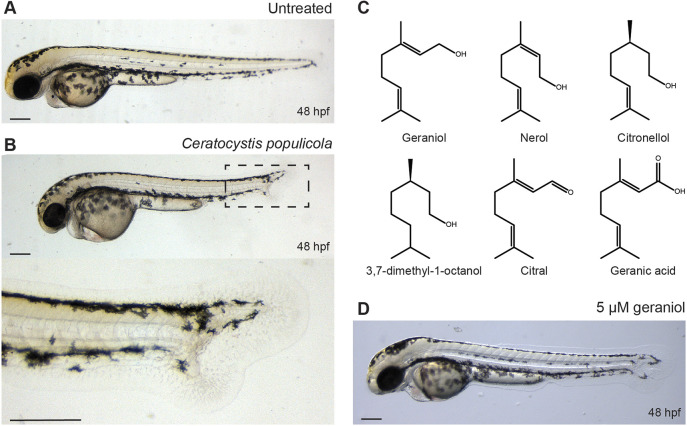
**Monoterpenes from *C. populicola* induce an ectopic tail.** (A) Untreated control (48 hpf). (B) An example of ectopic tail phenotype induced by the filtrate of *C. populicola*, treatment 6-48 hpf. (C) Molecular structures of compounds inducing ectopic tail. (D) An example of phenotype induced by 5 µM geraniol, treatment 8-24 hpf. Scale bars: 200 µm.

Previously, we reported a phenotypic screen of 10,207 fungal filtrates on developing zebrafish embryos (Hoeksma et al., 2019). The filtrate of the fungus *C. populicola* (CBS 119.78) was found to induce ectopic tail and fin formation on the ventral side of zebrafish embryos, accompanied by a split notochord and a shorter body axis compared to untreated embryos at 48 h post fertilization (hpf) (Fig. 1A,B). We selected this fungus for purification and identification of the active compound(s) and to examine the phenotype further.

As *Ceratocystis* species are known to produce a wide range of volatile compounds (Lanza and Palmer, 1977; Sanchez et al., 2002; Sprecher and Hanssen, 1983), we attempted to purify the active compound by distillation (Fig. S1A) and tested the resulting fractions on zebrafish embryos. The most potent fraction was analyzed first by liquid chromatography-mass spectrometry (LC-MS) revealing a distinct fragmentation pattern specific for the active compound at m/z=137.2, 95.1 and 81.1 (Fig. S1B). High resolution mass spectrometry revealed a monoisotopic mass of 177.1255 (M+Na^+^), resulting in a likely molecular formula of C_10_H_18_O (177.1256, calculated for C_10_H_18_ONa^+^).

Various monoterpene alcohols match this molecular formula of C_10_H_18_O and distinct mass spectrometry fragmentation pattern (Holzinger et al., 2000; Steeghs et al., 2007). Moreover, monoterpene alcohols are known to be produced by *Ceratocystis* species (Sprecher and Hanssen, 1983). Therefore, we obtained a selection of commercially available monoterpene alcohols (Fig. 1C) and tested them on zebrafish embryos in serial dilutions for their ability to induce ectopic tails (Fig. S2). Of the monoterpene alcohols, geraniol showed the best capacity to induce ectopic tails at concentrations as low as 2 µM (Fig. S2A). Throughout this paper we used 5 µM, because it reliably induced ectopic tails (Fig. 1D). Nerol, a stereoisomer of geraniol, was slightly less potent, with a minimal inducing concentration (MIC) of 5 µM (Fig. S2B). In contrast, linalool and cyclic monoterpene alcohols such as eucalyptol and α-terpineol were not able to induce ectopic tails up until a concentration of 500 µM. In addition, we also tested closely related monoterpenes, such as geranic acid (C_10_H_16_O_2_), the carboxylated form of geraniol, which was able to induce ectopic tails at a lowest concentration of 1 µM (Fig. S2C). Other closely related compounds such as 3,7-dimethyl-1-octanol (C_10_H_22_O), citral (C_10_H_16_O) and citronellol (C_10_H_20_O) (Fig. 1C) were also able to induce ectopic tails, albeit at 100 µM, 25 µM and 25 µM, respectively (Fig. S2D-F). All compounds that induced ectopic tails induced increasingly severe pleiotropic phenotypes at higher concentrations. In conclusion, we identified monoterpene alcohols, particularly geraniol, as the active compounds from *C. populicola* that induced ectopic tail formation in zebrafish embryos and, in the process, we identified chemically related monoterpenes to induce ectopic tails as well.

### Geraniol-induced ectopic tail formation is zebrafish strain dependent

**Fig. 2.**
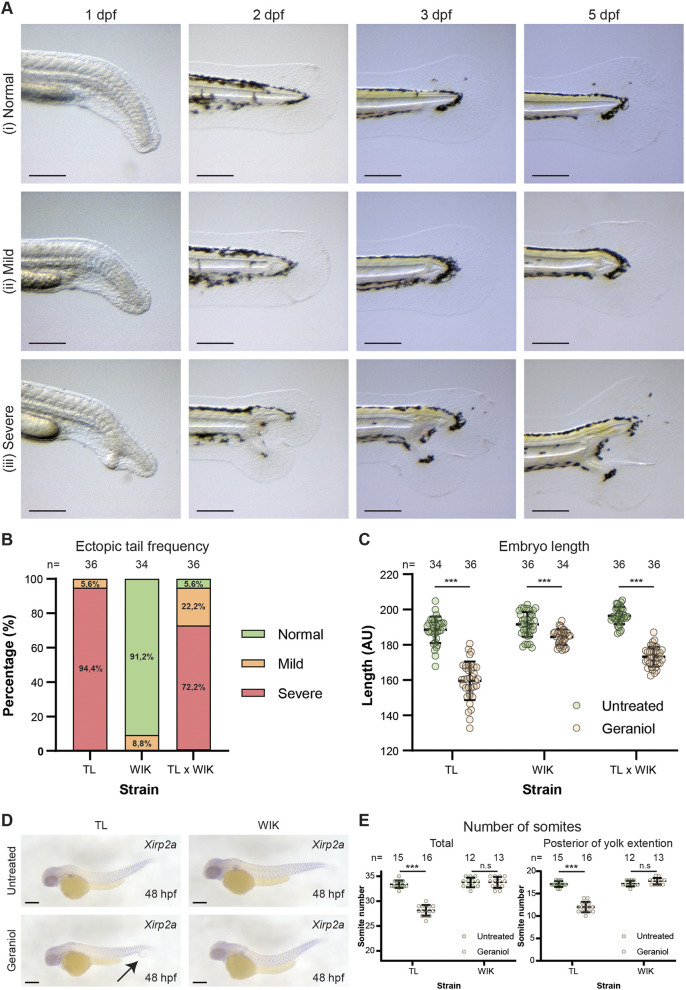
**Geraniol induced an ectopic tail in TL but not WIK embryos.** (A) Variations of phenotypes caused by 5 µM geraniol (treatment 8-24 hpf). Representative examples of a (i) normal, (ii) mild and (iii) severe embryo, imaged at the indicated times, are shown. (B) Phenotype frequency per zebrafish strain using TL and WIK family crosses and single crosses of TL with WIK. Total number of embryos (*n*) is indicated for each strain. (C) Quantification of zebrafish embryo length in arbitrary units at 48 hpf per zebrafish strain, untreated and 5 µM geraniol-treated as indicated. Results are expressed as mean±s.d. Significance was determined using an unpaired two-tailed *t*-test (***P<0.001). Total number of embryos (*n*) per condition is indicated. (D) Expression pattern of *xirp2a* in untreated and geraniol-treated (5 µM, 8-24 hpf) TL and WIK embryos, fixed at 48 hpf was established by *in situ* hybridization and imaged. Representative embryos are shown. (E) The total number of somites was counted as well as the number of somites posterior to the yolk extension. Average number of somites ±s.d. is shown. Significance was determined using an unpaired two-tailed *t*-test (***P<0.001). n.s, not significant. Scale bars: 200 µm.

To investigate the morphological defects after 48 hpf, we treated embryos with 5 µM geraniol from 8 until 24 hpf and tracked tail development until 5 dpf. Variable tail phenotypes were observed, which were classified in three distinct categories (Fig. 2A): (1) normal tail development, (2) mild, the notochord was split at the tail tip, but no ectopic tissue was formed, and (3) severe, tails had ectopic tissue and a split notochord. Strikingly, distinct responses to geraniol treatment were observed when using different zebrafish strains. When using TL embryos, 94.4% were found to develop a severe phenotype upon geraniol treatment, with the remaining 5.6% developing a mild phenotype (Fig. 2B). Overall, treated TL embryos were on average 15.3% shorter than untreated embryos (Fig. 2C). In contrast, 91.2% of geraniol-treated Wild Indian Karyotpe (WIK) embryos showed normal tail morphology, no embryos exhibited the severe phenotype and only 8.8% displayed a mild phenotype. Overall, treated WIK embryos were slightly shorter (3.7%) than untreated embryos. While higher concentrations of geraniol did not increase the number of ectopic tails in WIK embryos, a pleiotropic phenotype was observed, similar to the high geraniol-induced phenotype observed in TL embryos. Finally, treatment of embryos from crosses of TL with WIK resulted in fewer ectopic tails (72.2%) and on average the embryos were 11.9% shorter.

To investigate the differences in embryo length upon geraniol treatment in more detail, *in situ* hybridization was performed using a *xirp2a* probe (Fig. 2D), which marks the somite boundaries. Geraniol-treated TL embryos, but not WIK embryos, developed significantly fewer somites than untreated embryos (Fig. 2E). This difference was caused by a reduced number of somites posterior to the yolk extension. Furthermore, in both TL and WIK embryos treated with geraniol, the width of the somites was reduced posterior to the yolk extension compared to untreated embryos, whereas there were no differences in the width of anterior somites. In addition, *xirp2a* expression was observed in ectopic tails, indicating that somites are formed in ectopic tails. In conclusion, geraniol treatment induced ectopic tails and affected somite formation in a zebrafish strain-dependent manner.

### Ectopic tail formation initiates at tail bud stage

**Fig. 3.**
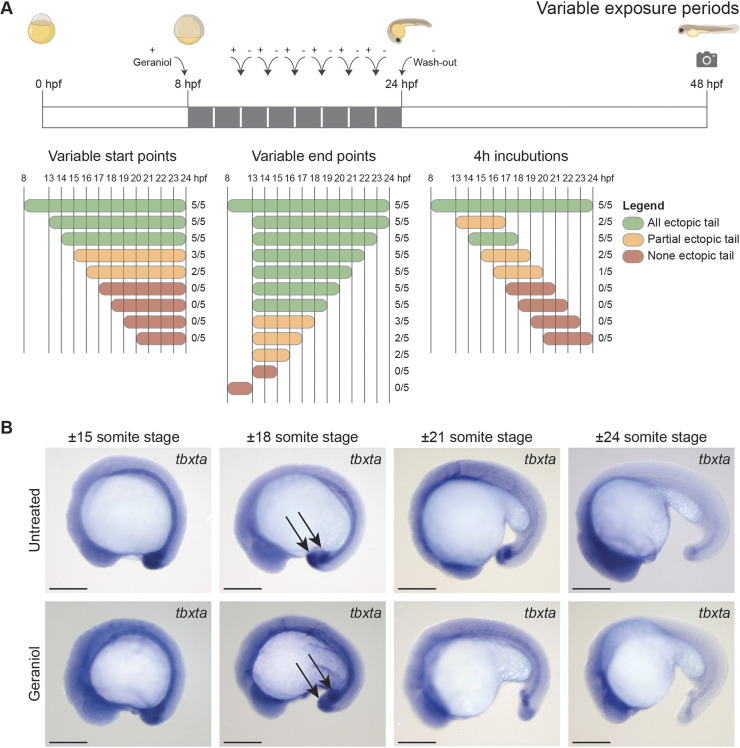
**Embryos are sensitive to geraniol between 14 and 18 hpf and geraniol treatment affects *tbxta* expression.** (A) Embryos treated with 5 µM geraniol for variable incubation periods (five embryos per condition). Embryos were washed twice with E3 after treatment. Phenotypes were scored at 48 hpf. Green indicates that all embryos developed an ectopic tail, orange indicates that one to four embryos developed an ectopic tail, red indicates that no embryos developed an ectopic tail. The actual number of embryos that developed an ectopic tail of the total number of embryos is indicated. (B) *In situ* hybridization showing *tbxta* expression at 15- to 24-somite stage as indicated. Control (untreated) and embryos treated with 5 µM geraniol from 8 hpf onwards are shown. Arrows highlight the two *tbxta*-expressing buds at the 18-somite stage in both untreated and geraniol-treated embryos. Scale bars: 200 µm.

To determine at which developmental stages geraniol induced ectopic tail formation, we tested geraniol treatment on TL zebrafish embryos for various incubation periods (Fig. 3A). Treatments starting at or before 14 hpf induced ectopic tails in all embryos, whereas starting at either 15 or 16 hpf only induced ectopic tails in 60% and 40% of the embryos, respectively. Treatment starting at 17 hpf or later did not induce any ectopic tails. Conversely, treatment until 18 hpf or beyond induced ectopic tails in all embryos, whereas treatment until 15, 16 or 17 hpf only induced ectopic tails in part of the embryos and aborting treatment at earlier time points did not induce any ectopic tails. Taken together, incubating embryos with geraniol between 14 and 18 hpf was necessary and sufficient for development of an ectopic tail. This period includes the tail bud stage at ∼16 hpf (Kimmel et al., 1995).

Next, the morphological events underlying the onset of the phenotype were investigated. Geraniol-treated and untreated embryos were fixed at various time points between ∼15-somite stage and 24-somite stage. *In situ* hybridization targeting the tail bud and notochord marker *tbxta* (formerly known as *ntl*) showed no apparent difference at the 15- and 18-somite stage between treated and untreated embryos (Fig. 3B). Notably, at 18-somite stage *tbxta* appeared to be expressed in two distinct buds in both treated and untreated embryos, with the anterior bud co-localizing with Kupffer's vesicle. It is noteworthy that *tbxta* plays an essential role in maintaining neuromesodermal progenitors (Martin and Kimelman, 2008). The observation that *tbxta* was expressed in two distinct buds even in untreated embryos might indicate that there are two separate populations of progenitor cells that give rise to the tail. At the 21- and 24-somite stage a single bud remained in untreated embryos, whereas in treated embryos the two distinct buds persisted, and a split notochord was observed. This suggests that failure to merge the two *tbxta*-positive pools resulted in ectopic tail formation in geraniol-treated embryos.

### Geraniol uses a distinct mechanism compared to other known ectopic-tail-inducing compounds

Previously, several other small molecules, such as pCAME and BMP-inhibitors dorsomorphin, DMH-1 and cercosporamide, were reported to induce ectopic tail tissue (Gebruers et al., 2013; Hoeksma et al., 2020; Yang and Thorpe, 2011). The effect of these compounds was compared by testing them in parallel to geraniol in our assay (Fig. S3) and distinct defects were found. TL-embryos treated with DMH-1 showed a dorsalized phenotype lacking parts of the ventral fin, a phenotype we never observed in geraniol-treated embryos, with only a small percentage of the embryos developing ectopic tissue (Fig. S3A). Using pCAME, we only observed ectopic tails upon 1 h pulse treatments at 100 µM, as previously reported (Gebruers et al., 2013), but not upon continuous incubation between 8 and 24 hpf (Fig. S3B). Furthermore, to establish whether there is any synergistic effect between these compounds and geraniol, we performed combination treatments using half the MIC and/or the full MIC of the compounds. Treatment with a combination of geraniol and DMH-1 each at full MICs (2 µM and 250 nM, respectively) resulted in a more severe phenotype, but no increase in the frequency of ectopic tail formation (Fig. S4A). Also, no synergy was observed nor additive defects using half the MIC for geraniol and pCAME (Fig. S4B).

WIK embryos were less sensitive to geraniol. Yet, incubation of WIK embryos with BMP-inhibitor DMH-1 induced loss of ventral fins at a frequency similar to TL-embryos (Fig. S5), indicating that WIK embryos were sensitive to DMH-1. Taken together, geraniol appears to induce ectopic tail formation through a different mechanism than DMH-1 and pCAME.

### Key regulators of retinoic acid signaling are downregulated in tail buds of geraniol-treated embryos

To uncover the molecular mechanisms underlying the onset of the ectopic tail, whole transcriptome mRNA sequencing was performed on individual tail buds from geraniol-treated or untreated 15-somite stage embryos. Before differential gene expression analysis, the sequencing depth of each sample was checked by determining the number of reads per gene and total read number (Fig. S6A). We excluded three samples from differential gene expression analysis due to low quality.

**Fig. 4.**
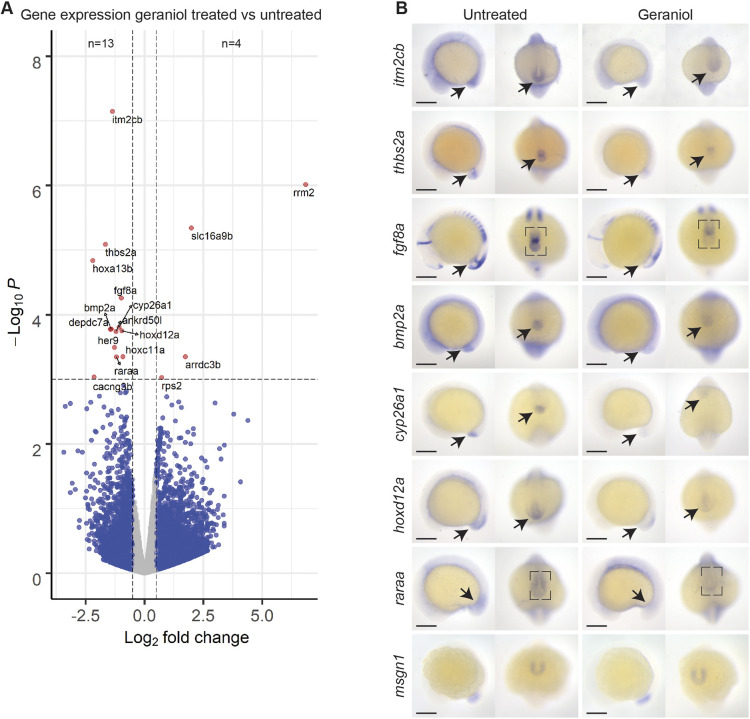
**Differential gene expression in response to geraniol treatment.** (A) RNA was extracted from the tip of the tail of 15-somite-stage embryos untreated (*n*=7) or treated with 5 µM geraniol from 8 hpf onwards (*n*=6) and processed for bulk RNA-sequencing. Volcano plot shows differentially expressed genes (total genes included=23,432) between geraniol-treated and control embryos. Genes with a fold change of at least ±0.5 and a *P*-value lower than 0.001 were scored as positive. (B) *In situ* hybridization of selected differentially expressed genes and *msgn1*, imaged laterally and posteriorly. Representative untreated and 5 µM geraniol-treated (from 8 hpf onwards) embryos are shown. Scale bars: 200 µm.

We then performed differential gene expression analysis and found 13 significantly downregulated and four significantly upregulated genes in the tail bud of geraniol-treated embryos compared to untreated (Fig. 4A, Fig. S6B). Notably, downregulated genes included *cyp26a1*, *fgf8a*, *raraa* and several hox genes, *hoxa13b*, *hoxd12a* and *hoxc11a*, which are all genes that are known for their involvement in retinoic acid signaling (Shimizu et al., 2006; Ye and Kimelman, 2020). More specifically, *cyp26a1* encodes an enzyme that metabolizes retinoic acid, *fgf8a* encodes an FGFR ligand, and its expression is regulated by retinoic acid signaling (Hamade et al., 2006). *Raraa* encodes Retinoic Acid Receptor Aa, a retinoic acid-responsive transcription factor. Expression of *hoxa13b*, *hoxd12a* and *hoxc11a* is directly controlled by retinoic acid signaling. Moreover, *hoxa13b* is known for its role in mesoderm formation and axis elongation (Ye and Kimelman, 2020). Interestingly, the two most significantly downregulated genes, *itm2cb* and *thbs2a*, have not been associated with zebrafish tail development. Next, the localization of differential gene expression was assessed by *in situ* hybridization using 15-somite-stage embryos (Fig. 4B). In agreement with the RNA-sequencing results, lower staining intensity was observed for most of the downregulated genes. Furthermore, *itm2cb* and *thbs2a* were specifically expressed in the tail bud and Kupffer's vesicle, respectively, which implicates their involvement in tail development, which has not been reported before. We used *msgn1*, a regulator of paraxial mesoderm differentiation of neuromesodermal stem cells, as a control (Manning and Kimelman, 2015; https://zfin.org/ZDB-PUB-040907-1). *Msgn1* is highly expressed in the tail bud and did not show differential expression using either RNA-sequencing or *in situ* hybridization (Fig. 4B).

*In situ* hybridization using probes for these differentially expressed genes in embryos from WIK incrosses did not show clear differences in expression, whereas quantitative real-time PCR only showed slightly decreased expression of *fgf8a* and increased expression of *itm2cb* between geraniol-treated and untreated embryos (Fig. S7). This underlines the strain-specific effect of geraniol.

Differentially downregulated genes were implicated in retinoic acid signaling. Geraniol has chemical resemblance with retinol with its polyene hydrophobic tail. Interestingly, citral, the aldehyde form of geraniol, has been found to interfere with retinoic acid signaling in *Xenopus* (Schuh et al., 1993). Therefore, we interrogated the potential role of retinoic acid and retinol in ectopic tail formation. Consistent with previous reports, retinoic acid (50 nM) and retinol (5 µM) induced bent tails (Fig. S8A), but not an ectopic tail, as observed with geraniol (D'Aniello et al., 2015; Minucci et al., 1996). Co-incubation of retinol or retinoic acid with geraniol also did not rescue ectopic tail formation (Fig. S8A). Ectopic tails were still established in addition to the retinoid induced phenotype. Furthermore, *in situ* hybridization (Fig. S8B) and real-time PCR (Fig. S8C,D) indicated upregulation of *cyp26a1* in response to retinoic acid and retinol treatment, contrary to geraniol treatment, whereas *hoxd12a* was severely downregulated in response to treatment with retinoic acid or retinol (Fig. S8C,D). Co-incubation of geraniol with either retinoic acid or retinol still resulted in elevated *cyp26a1* levels compared to control conditions. To investigate a potential causal role, *cyp26a1* was overexpressed by micro-injection of synthetic mRNA encoding *cyp26a1* at the one-cell stage. Ectopic expression of *cyp26a1* did not rescue geraniol treatment, suggesting that reduced *cyp26a1* expression did not cause ectopic tail formation. Although factors of the retinoic acid signaling pathway were downregulated in response to geraniol, altered retinoic acid signaling did not induce ectopic tail formation.

### Left-right asymmetry is not affected by geraniol treatment

In order to determine the cause of ectopic tail formation, the emergence of ectopic tails in geraniol-treated embryos was studied. Ectopic tails initiate at the site where Kupffer's vesicle normally collapses and disappears. Kupffer's vesicle is a transient liquid filled spheroid structure located on the ventral and posterior side of the zebrafish embryo, and plays a crucial role in the establishment of left-right asymmetry in the embryo (Essner et al., 2005; Kupffer, 1868). To see if geraniol affects left-right asymmetry, *in situ* hybridization was performed using the cardiomyocyte marker *myl7* and the liver marker *fabp10a*. No left-right asymmetry defects were apparent in the heart or the liver in response to geraniol treatment (Fig. S9). Although there was no difference in liver left-right asymmetry, the liver size in geraniol-treated embryos appeared to be reduced at 48 hpf. However, at 5 dpf there was no longer a difference in liver size detected, suggesting that geraniol treatment induced a transient growth delay of the liver. In conclusion, geraniol does not affect Kupffer's vesicle-mediated left-right asymmetry.

### Epithelial-to-mesenchymal transition is affected in Kupffer's vesicle epithelial cells upon geraniol treatment

Previously, it has been shown that upon Kupffer's vesicle collapse, KVDCs undergo epithelial-to-mesenchymal transition (EMT) (Amack, 2021; Ikeda et al., 2022). To study the involvement of KVDCs in ectopic tail formation, we performed time-lapse imaging using two transgenic lines: *Tg(sox17:EGFP)*, which marks endodermal and Kupffer's vesicle epithelial cells (Sakaguchi et al., 2006), and *Tg(dand5:EGFP)*, which exclusively marks Kupffer's vesicle epithelial cells (Ikeda et al., 2022).

**Fig. 5.**
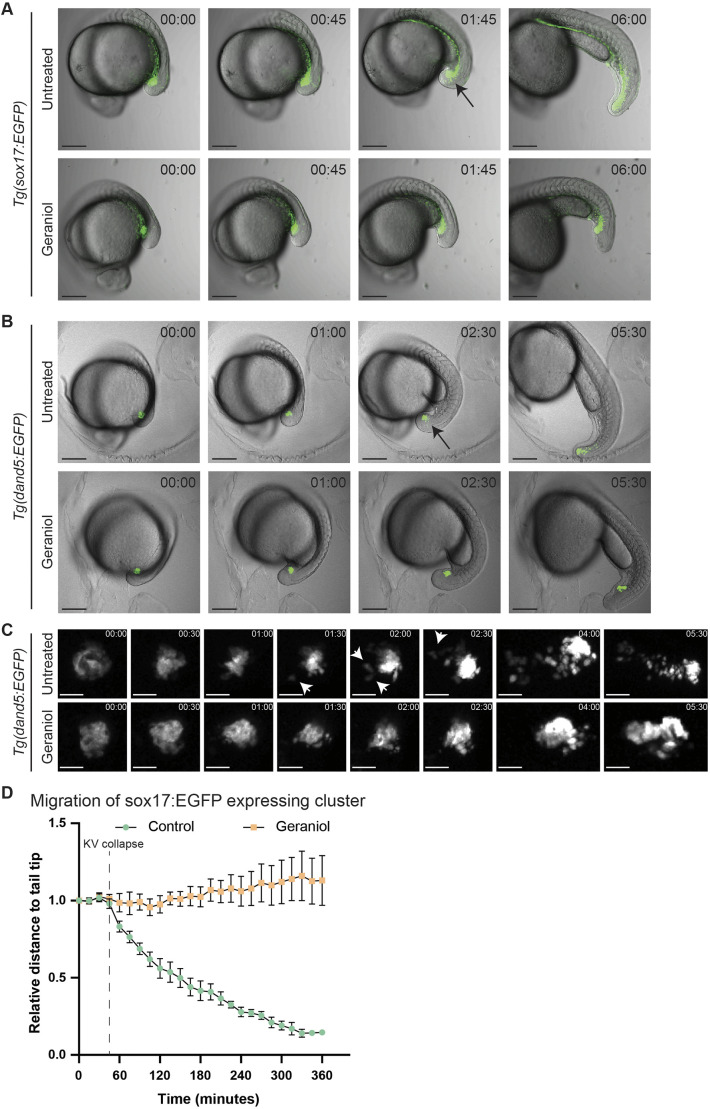
**Posterior migration of Kupffer's vesicle epithelial cells after Kupffer's vesicle collapse is impaired in response to geraniol treatment.** (A) Stills from time-lapse imaging (Movie 1) of control (untreated) and geraniol-treated (5 µM from 8 hpf onwards) *Tg(sox17:EGFP)* embryos. (B) Stills from time-lapse imaging (Movie 2) of control (untreated) and geraniol-treated (5 µM from 8 hpf onwards) *Tg(dand5:EGFP)* embryos. In A and B, 00:00 (hh:mm) corresponds approximately to developmental 12-somite stage. Arrows indicate Kupffer's vesicle at the time of its collapse. (C) Magnification of stills from time-lapse imaging using *Tg(dand5:EGFP)* embryos, showing Kupffer's vesicle collapse in both untreated and geraniol-treated embryos (00:00-01:30) and migration of Kupffer's vesicle epithelial cells. The arrows indicate migratory behavior of fluorescent cells following Kupffer's vesicle collapse in untreated embryos (01:30-05:30). (D) Quantification of posterior cell cluster migration during time-lapse imaging of *Tg(sox17:EGFP)* embryos. Each point in the graph represents the mean distance to the tip of the tail, relative to the distance at the start of the movie. Error bars represent s.e.m. *n*=5 for both untreated and geraniol-treated embryos. Scale bars: 200 µm (A,B); 50 µm (C).

We imaged embryos every 15 min from before the onset of tail formation, at ∼10- to 12-somite stage, until ∼24 hpf. Around the 14-somite stage, in untreated embryos, GFP-positive Kupffer's vesicle epithelial cells in both *Tg(sox17:EGFP)* and *Tg(dand5:EGFP)* reporter lines converged inwards into the lumen of Kupffer's vesicle, after which Kupffer's vesicle disintegrated and ceased to exist (Fig. 5A-C, Movies 1,2). Directly after the collapse of Kupffer's vesicle, the KVDCs transitioned from an epithelial state into a migratory state, a process known as EMT. Cells started to dissipate from the main cluster of KVDCs, as indicated by the arrows in Fig. 5A-C. In *Tg(sox17:EGFP)* embryos this was reflected in a sprouting pattern upon Kupffer's vesicle-collapse. In addition, the main cluster of GFP-positive cells migrated towards the tip of the tail, which we quantified by measuring the proximal distance of the fluorescent signal to the tip of the tail (Fig. 5D). When treated with geraniol, Kupffer's vesicle did disintegrate, but following the collapse, the KVDCs failed to undergo EMT, did not disperse and did not migrate to the tail. The proximal distance of *sox17:EGFP*-positive cells to the tip of the tail remained equal throughout tail development (Fig. 5D) and an ectopic tail formed at the former Kupffer's vesicle site.

Similarly, in untreated *Tg(dand5:EGFP)* embryos, cells dispersed from the main cluster after Kupffer's vesicle-collapse, and the KVDCs dispersed and migrated towards the tail tip (Movie 2, Fig. 5B,C). However, in geraniol-treated *Tg(dand5:EGFP)* embryos, the fluorescent cells did not disperse after Kupffer's vesicle collapse, and they did not contribute to the main tail. The location of the fluorescent cells in geraniol-treated embryos coincided with the site of ectopic tail formation.

**Fig. 6.**
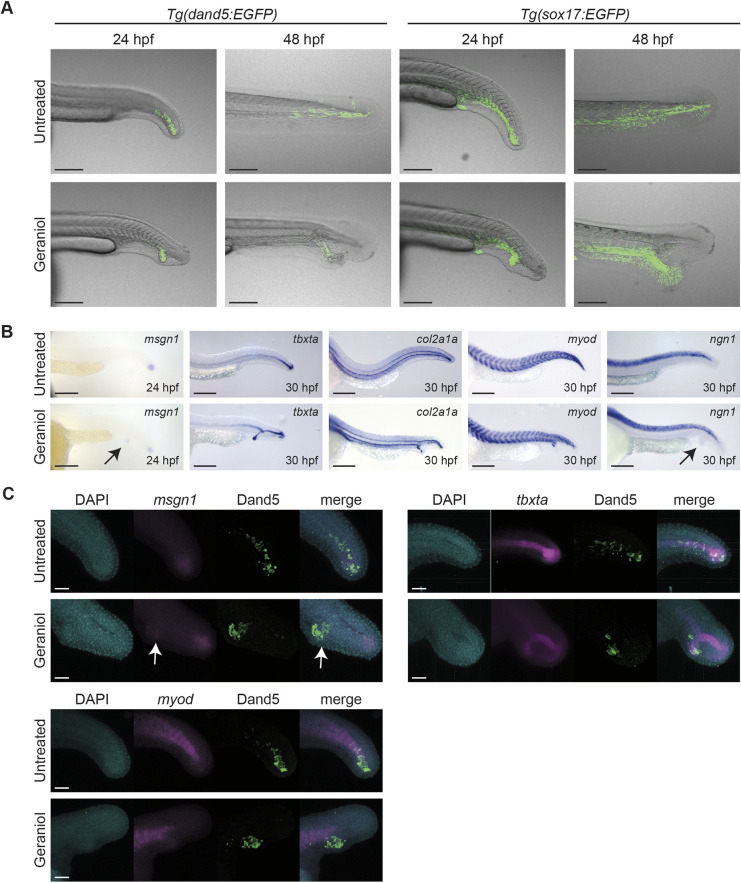
**All cell fates except neural tissue contribute to ectopic tail.** (A) Representative embryos showing expression of *dand5:EGFP* and *sox17:EGFP* at 24 hpf and 48 hpf. Control (untreated) and embryos treated with 5 µM geraniol from 8-24 hpf are depicted. (B) Control (untreated) and treated (5 µM geraniol from 8-24 hpf) embryos were fixed at 24 hpf for *msgn1*, and 30 hpf for the other markers and processed for *in situ* hybridization using various markers. Arrows indicate the ectopic tail. (C) Fluorescent *in situ* hybridization targeting various markers as indicated, combined with DAPI staining and immunohistochemistry targeting GFP in *tg(dand5:EGFP)* embryos. Arrows indicate co-expression of *msgn1* and Dand5 in KVDCs of geraniol-treated embryos. Scale bars: 200 µm.

Subsequently, expression of *dand5* and *sox17* was evaluated in TL embryos at 24 hpf and 48 hpf. In untreated embryos, GFP-positive cells from both *Tg(dand5:EGFP)* and *Tg(sox17:EGFP)* transgenes were located ventrally to the notochord at 24 hpf and were more dispersed throughout the posterior end of the tail at 48 hpf. However, in treated *dand5:EGFP* embryos, expression was strictly confined to the ectopic tail and expression of *sox17:EGFP* did not extend into the main tail posterior to the notochord split (Fig. 6A). Interestingly, in transgenic *Tg(dand5:EGFP)* embryos in the WIK background, GFP-positive KVDCs migrated normally towards the posterior end of the tail after geraniol treatment (Fig. S10). Hence, the block in KVDC migration is specific for TL embryos.

### The severity of tail and notochord defects correlates with the extent of KVDC dispersion

The effect of DMH-1 on *dand5:EGFP* embryos was tested and variable effects were detected in the distribution of GFP-positive cells at 24 and 48 hpf (Fig. S11). In embryos that developed an ectopic tail, most of the GFP-positive KVDCs located to the ectopic tail, and a small fraction located to the main tail (Fig. S11C,D). In other embryos, a slightly larger fraction of the KVDCs migrated to the tail tip, and these embryos did not develop an ectopic tail (Fig. S11E-H). Alternatively, KVDCs were distributed in distinct clusters along the ventral axis (Fig. S11I,J), or the KVDC distribution appeared to be normal (Fig. S11K,L), in which cases no ventral fin was formed and no ectopic tail developed. Notably, embryos with disturbed KVDC distribution all showed notochord defects including either kinked, bifurcated, interrupted or narrowed notochords. Overall, the severity of the notochord phenotype induced by DMH-1 treatment appeared to correlate with the distribution of KVDCs. Yet, loss of the ventral fin did not appear to correlate with KVDC distribution. Only when most of the KVDCs did not migrate following DMH-1 treatment, an ectopic tail formed.

### Progenitor marker genes are expressed in ectopic tails

Previously, it has been shown that KVDCs contribute to notochord and somite tissue (Ikeda et al., 2022). We performed *in situ* hybridization to determine which cell types are present in ectopic tails (Fig. 6B). In geraniol-treated embryos *msgn1*, regulator of paraxial presomitic mesoderm differentiation, was expressed in two distinct clusters, one in the normal and one in the ectopic tail at 24 hpf. This indicates that two separate clusters of presomitic mesoderm and tail progenitor cells exist in embryos with ectopic tails. In addition, other markers such as notochord marker *tbxta*, somite and muscle progenitor marker *myod*, and hypochord marker *col2a1a* were expressed in the ectopic tail as well as the regular tail (Fig. 6B). However, whereas dorsal *col2a1a* expression appeared as an uninterrupted line, ventral *col2a1a* expression was interrupted and appeared to deviate into the ectopic tail in treated embryos. Notably, ectopic tails lacked expression of neural ectoderm marker *ngn1* (*neurog1*), which is only dorsally expressed in the embryo, suggesting that ectopic tails lack the capacity or do not receive essential cues to form neural tissue.

To assess whether KVDCs contribute to notochord and somite tissue in the ectopic tail, we performed fluorescent *in situ* hybridization in combination with immunofluorescence in *Tg(dand5:EGFP)* embryos (Fig. 6C). In both control and geraniol-treated embryos, several cells were found co-expressing *dand5*-driven expression of EGFP and *msgn1*, *tbxta* or *myod*, indicating that also in ectopic tails KVDCs are contributing to various tissues.

In conclusion, the imaging and *in situ* hybridization experiments revealed that geraniol treatment blocked migration of KVDCs towards the tip of the tail. Instead, these cells contributed to an ectopic tail bud, which expressed all markers of the normal tail, except the neural marker, *ngn1*. Under normal conditions – in the absence of geraniol – the KVDCs migrate towards the posterior and contribute to the developing tail.

### Kupffer's vesicle ablation through *tbxta*-MO injection rescues ectopic tail formation

**Fig. 7.**
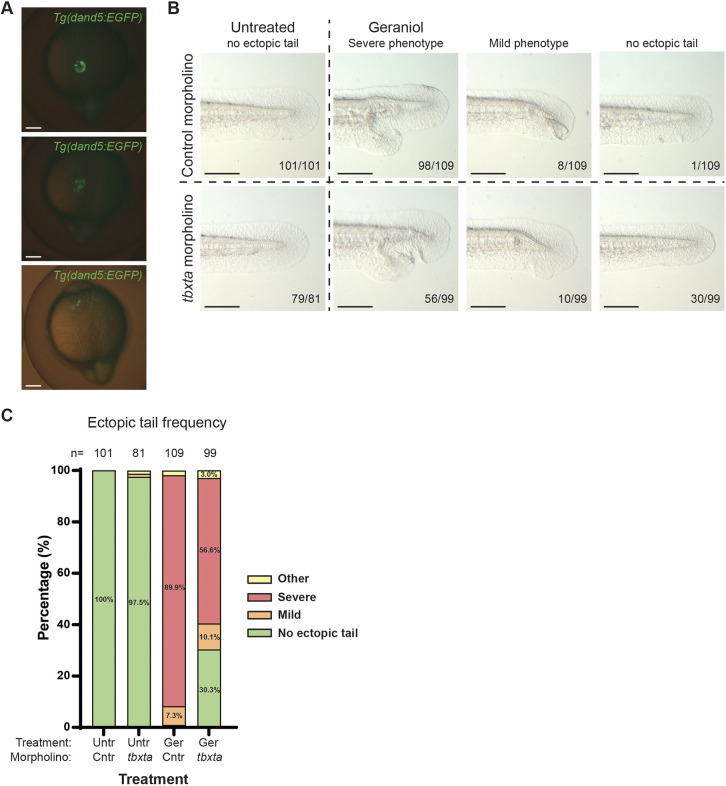
***Tbxta* morpholino injections rescued ectopic tail formation.** (A) Phenotypes of *tbxta*-morpholino injected *Tg(dand5:EGFP)* embryos at ∼15 somite stage. (B) Phenotypes of morpholino-injected, control and *tbxta* embryos, and treated embryos at ∼48 hpf. (C) Phenotype frequency of morpholino-injected treated and untreated embryos at 48 hpf.

Finally, to verify the importance of KVDCs in ectopic tail formation, we performed Kupffer's vesicle ablation experiments by injecting embryos at ∼512- to 1024-cell stage with *tbxta*-MO or a control MO as described before (Amack and Yost, 2004; Amack et al., 2007). Previously, it has been shown that *tbxta*-MO injections at later stages result in chimeric knockdown embryos, specifically targeting dorsal forerunner cells, thus affecting morphogenesis of Kupffer's vesicle in a fraction of the embryos (Amack and Yost, 2004). We performed *tbxta*-MO injections in *tg(dand5:EGFP)* embryos and observed a variation of phenotypes at the 15-somite stage, ranging from embryos with a reduced number of disorganized *dand5:EGFP*-positive cells to apparently normal Kupffer's vesicle development (Fig. 7A). Next, MO-injected embryos were treated with geraniol or left untreated and scored for ectopic tail formation (Fig. 7B). In line with previous experiments, geraniol treatment induced ectopic tail formation in 89.9% of control-MO injected embryos and a mild phenotype in 7.3% of the embryos (Fig. 7C). Strikingly, geraniol treatment did not induce an ectopic tail in 30.3% of the *tbxta*-MO injected embryos and induced a severe phenotype in only 56.6% of the embryos. Taken together, the results indicate that KVDCs are essential for ectopic tail formation upon geraniol treatment.

## DISCUSSION

Here, we report that monoterpene alcohols, in particular geraniol, from the fungus *C. populicola* induced ectopic tail formation in TL zebrafish embryos during tail bud stage. Treatment with geraniol affected retinoic acid signaling, however the precise role of this pathway in ectopic tail formation remained unclear. We show that migration of KVDCs was affected in geraniol-treated embryos, resulting in prolonged separation of two progenitor pools and eventually ectopic tail formation, which was rescued by Kupffer's vesicle ablation by *tbxta*-MO injection at the mid-blastula stage.

Previously, ectopic tail formation has mostly been associated with effects on BMP-signaling and non-canonical Wnt-signaling (Gebruers et al., 2013; Yang and Thorpe, 2011). Our results show that geraniol-induced ectopic tail formation was not due to BMP-signaling, because we did not observe any synergistic effects of treatments combining geraniol with DMH-1 or pCAME. In addition, the BMP-signaling inhibitor, DMH-1, had a similar penetrance in embryos from both TL and WIK strains, whereas geraniol only induced ectopic tails in TL embryos.

The difference in effect of geraniol on TL and WIK strains (Fig. 2) may provide an opportunity to deduce the targets or pathways involved in ectopic tail formation by geraniol. Genetic comparison of TL and WIK strains may provide clues regarding the targets of geraniol. It is well-established that extensive genetic variations exist between these two wild-type strains, which may affect the outcome of experiments, including drug screens (Guryev et al., 2006). It is likely that the target of geraniol is either differentially expressed between WIK and TL strains or harbors single nucleotide polymorphisms (SNPs), which may influence binding or conversion of geraniol. Transcriptomic or genomic approaches could lead to identification of differences between WIK and TL strains and flag potential targets of geraniol.

Based on our RNA expression data (Fig. 4), retinoic acid signaling appeared to be a good candidate pathway for involvement in ectopic tail formation. Furthermore, in WIK embryos we did not observe differential expression of retinoic acid signaling genes (Fig. S7), which would be consistent with the lack of geraniol-induced ectopic tail formation in WIK embryos. Moreover, citral, the aldehyde form of geraniol, was previously reported to interfere with retinoic acid signaling in *Xenopus* (Schuh et al., 1993). Geraniol has chemical resemblance to retinol with its polyene hydrophobic tail, and disruption of the retinoic acid balance in the zebrafish tail was reported to cause an array of tail phenotypes (Rydeen et al., 2015). However, whereas expression of *cyp26a1* was downregulated by geraniol treatment, it was upregulated by retinoic acid, even when co-treated with geraniol. Upon co-treatment with geraniol and retinoic acid, ectopic tails still formed, suggesting that retinoic acid was not directly involved in ectopic tail formation. Expression of *cyp26a1* and *fgf8a* are also modulated by other factors such as Wnt and Notch signaling and cdx-genes (Draut et al., 2019). Potentially, differential expression of other genes that we identified and that have previously been associated with EMT in other contexts has a causal role. For example, *arrdc3b* is upregulated in response to geraniol (Fig. 4A). ARRDC3 has recently been reported to inhibit EMT in triple knockout breast cancer and has tumor suppressing properties (Soung et al., 2019). However, the function of ARRDC3 remains to be determined definitively. The mouse homolog of *thbs2a*, *Thbs2*, is reported to be involved in cell adhesion and migration of mesenchymal cells in mouse (Kyriakides et al., 1998), and human *THBS2* has been reported to be a tumor suppressor (Liu et al., 2023). The mouse homolog *Itm2c*, an integral transmembrane protein, has been reported to function in the brain (Choi et al., 2001), and recently human *ITM2C* has been associated with colorectal cancer (Maurya et al., 2023). It will be interesting to investigate the role of these differentially expressed genes in tail morphogenesis and ectopic tail formation in the future, which may provide insights into their role in disease.

Our various timecourse treatments and time-lapse imaging experiments show that KVDCs had a crucial role in ectopic tail formation. Treatment with geraniol for 4 h during tail bud stage was sufficient to induce ectopic tails (Fig. 3A). This treatment window (14-18 hpf) coincided with the time that Kupffer's vesicle collapsed and that KVDCs started to migrate (Fig. 5C). Moreover, at tail bud stage we observed bifurcated expression of *tbxta* in untreated embryos (Fig. 3B), which was resolved during the next phase. In geraniol-treated embryos this bifurcated expression persisted (Fig. 3B). In addition, time-lapse imaging using *Tg(dand5:EGFP)* and *Tg(sox17:EGFP)* reporter lines revealed that this separation is most likely caused by inhibition of the transition into a migrative state (EMT) of KVDCs in response to geraniol treatment. Canonically, one single pool of neuromesodermal progenitor cells resides in the tailbud. The cells from this pool first undergo EMT and move into the maturation zone under the influence of *msgn1* and *tbx16*, and become migratory. Subsequently, mesodermal cells continue to move anteriorly through the presomitic mesoderm, after which they start to form somites and contribute to both mesodermal and neural lineages (Chalamalasetty et al., 2014; Manning and Kimelman, 2015). Our results suggest the existence of a separate cluster of progenitor cells originating from cells surrounding Kupffer's vesicle. Some KVDCs expressed progenitor markers such as *msgn1* and *tbxta* (Fig. 6B,C). At later stages, the ectopic tail of geraniol-treated embryos contained somite and notochord tissue, but not neural tissue (Figs 2D and 6B). This suggests that KVDCs only transdifferentiate into mesodermal lineages, not neural lineages, or potentially lack cues to differentiate into neural lineages. The extent of the plasticity of KVDCs is an interesting topic for future research.

Until recently, the fate of Kupffer's vesicle cells after the collapse of the Kupffer's vesicle was not well understood. Our data are consistent with recent reports that KVDCs undergo EMT and are able to transdifferentiate and contribute to various tissues in the zebrafish tail (Amack, 2021; Ikeda et al., 2022). Moreover, our results suggest that KVDCs were not merely being reemployed, but had an active role in tail morphogenesis. Perturbations in the localization and migration of KVDCs using geraniol resulted in the formation of ectopic tails (Figs 5 and 6). Treatment with the BMP inhibitor DMH-1 also affected the distribution of KVDCs and tail formation, albeit the defects were distinct from the geraniol-induced defects (Figs S3, S4, S11). Moreover, the severity of the defects directly correlated with the distribution of KVDCs: embryos with severely disrupted KVDC distribution displayed undulated or kinked notochords (Fig. S11E-J) or ectopic tails (Fig. S11C,D), whereas embryos with a normal distribution had normal notochords (Fig. S11K,L). Overall, interrupted migration or misplacement of KVDCs had a drastic impact on the organization of the tail and affected surrounding structures such as the notochord. Finally, when Kupffer's vesicle formation was blocked by MO-mediated knockdown of Tbxta expression, ectopic tail formation in response to geraniol was rescued, thus providing strong support for the role of KVDCs in ectopic tail formation and tail morphogenesis in general.

All results combined, we conclude that Kupffer's vesicle epithelial cells have transdifferentiation potential and are important for tail morphogenesis. Furthermore, Kupffer's vesicle harbors a cluster of tail progenitors, which are distinct from the caudal tail bud. Upon Kupffer's vesicle collapse, KVDCs become migratory and contribute to the patterning of the tail. Geraniol inhibits migration of KVDCs and thereby induces ectopic tail formation. In addition, altered distribution of KVDCs results in various notochord and tail defects. Overall, our results provide new insights into zebrafish tail morphogenesis and ectopic tail formation, and highlight the importance of KVDC migration and distribution.

## MATERIALS AND METHODS

### Embryo screening assay

For the initial experiments, embryos from family crosses of TL zebrafish were used. Fertilized eggs were first selected and washed with fresh E3-medium, before they were divided over 24-well plates with ten embryos per well in 1000 µl E3-medium. Samples were added in various concentrations and for various durations as mentioned in text and figure legends. Brightfield imaging was performed using either a Leica M165 FC microscope equipped with a Leica DMC5400 camera or a Leica MZFLIII microscope equipped with a Leica DFC320 camera while the embryos were mounted in 2% methyl cellulose. ImageJ was used for size and length measurements.

TL (ZDB-GENO-990623-2, #1174-TL) and WIK (ZDB-GENO-010531-2, #1171-WIK) zebrafish lines were originally obtained from EZRC (Germany). All procedures involving experimental animals were approved by the local animal experiments committee (Koninklijke Nederlandse Akademie van Wetenschappen-Dierexperimenten commissie) and performed according to local guidelines and policies in compliance with national and European law. Adult zebrafish were maintained as previously described (Aleström et al., 2019).

### Fungus culture and compound purification and identification

The fungus *C. populicola* (CBS 119.78) was grown on cornmeal agar (CMA) at 25°C for 7 days. Next, the agar with mycelium was cut into cubes of ∼5×5 mm, which were used to inoculate 20×100 ml bottles containing 50 ml Czepek dox broth+0.5% yeast extract. The medium was incubated at 25°C for 7 days, before filter sterilization using a 0.22 µm Millipore steritop filter. Approximately 125 ml of the fungal filtrate was used for vacuum distillation using the set-up as shown in Fig. S1A, using magnetic steering of the filtrate at 250 rpm and with heating increasing every 40 min: 50°C for 0-40 min; 75°C for 40-80 min; 150°C for 80-120 min. Samples were collected every 40 min.

Samples were analyzed using a LC-MS and high-resolution mass spectrometry (HRMS). HRMS was performed on a µQTOF instrument (Micromass), which was calibrated using sodium formate, followed by injection of the sample mixed with sodium formate inducing sodium adduct ions of the compound in the process.

### Live imaging

Eggs from either *Tg(sox17:EGFP)* or *Tg(dand5:EGFP)* crosses in a TL background were harvested and grown at 28.5°C until 8 hpf. Embryos were split over two 6 cm dishes, one dish was incubated with 5 µM geraniol (see Table S1 for chemicals and reagents), while the other was left untreated. Subsequently, the embryos were grown overnight at room temperature to delay growth. Fluorescent embryos were selected and placed in a glass-bottom dish or glass-bottom 24-well plate. *Tg(sox17:EGFP)* embryos were (co-)incubated with MS222/tricane during the experiment. *Tg(dand5:EGFP)* embryos were injected with bungarotoxin mRNA (Addgene plasmid #69542) at the one-cell stage to immobilize them. Embryos were imaged using a Leica SP8 microscope. *Z*-stacks were obtained every 15 min. Maximum projections and quantifications were created using ImageJ.

### *In situ* hybridization and immunostaining

Embryos were fixed in 4% paraformaldehyde either overnight at 4°C or for 3 h at room temperature. *In situ* hybridization was performed as previously described (Thisse and Thisse, 2008). Digoxigenin-labeled probes were generated from PCR products or plasmids. The sequences of the primers used to generate these PCR products are listed in Table S2. The reverse primers all contain a T7 RNA polymerase promoter site preceded by three bases to allow transcription of the PCR product. In situs were imaged using a Leica M165 FC microscope equipped with a Leica DMC5400 camera and a ring light. For fluorescent *in situ* experiments the NBT-BCIP reaction was replaced by Immpact Vector Red Substrate Alkaline Kit (VectorLabs, #SK-5105) according to the manufacturer's protocol, followed by immunostaining as previously described (Choorapoikayil et al., 2014) excluding the permeabilization step, using chicken anti-green fluorescent protein antibody (Aveslabs, #GFP-1010, 1:500; RRID:AB_2307313) as primary antibody and goat anti-chicken Alexa-488 as secondary antibody (Invitrogen, #A-11039, dilution 1:500; RRID:AB_2534096). Embryos were imaged using a Leica SP8 microscope and analyzed using ImageJ.

### RNA isolation from tail buds

Eggs from TL-crosses were harvested and grown at 28.5°C until 8 hpf. Embryos were split over two 6 cm dishes, one dish was incubated with 5 µM geraniol, while the other was left untreated. Subsequently, the embryos were grown overnight at room temperature to delay growth. At ∼15-somite stage the eggs were dechorionated and individual tails were isolated by cutting the tail bud using a BD Microlance 3 (30G×½″, #304000) needle. The tail buds were transferred to a 1.5 ml Eppendorf tube and immediately snap frozen in liquid nitrogen. RNA-extraction was performed using Trizol reagent according to the manufacturer's protocol using 1/10 of the volumes reported. The pellet was resuspended in 10 µl milli-Q water. The quality of the samples was assessed using a bioanalyzer. In total eight untreated and eight geraniol-treated samples were submitted for RNA-sequencing. Library preparation and RNA-sequencing was outsourced to Single Cell Discoveries. Differential gene analysis was performed using R-studio using R-library deseq2. Samples with a low number of reads were excluded from the analyses. The volcano plot has been generated using library EnhancedVolcano. Code used for analysis are available on request.

### Quantitative PCR

For quantitative PCR, five embryos per condition were pooled and snap frozen in liquid nitrogen. Subsequently, RNA-extraction was performed using Trizol reagent according to the manufacturer's protocol. Quantitative real-time PCR was performed using FastStart Universal SYBR Green Master Mix on a Bio-Rad CFX384 Touch Real-Time PCR Detection System with primers as mentioned in Table S3. For each condition, three biological replicates were used and in turn each gene was measured in either triplicate or quadruplicate. As internal reference housekeeping gene *actb1* was used. Analysis was performed using the Bio-Rad CFX Maestro software. Plots were generated using Prism GraphPad.

### Morpholino injections

MO experiments were performed as previously described (Amack and Yost, 2004; Amack et al., 2007). Briefly, unlabeled MOs against *tbxta* (5′-GACTTGAGGCAGGCATATTTCCGAT-3′) and a standard negative control (5′-CCTCTTACCTCAGTTACAATTTATA-3′) were purchased from GeneTools and injected in the yolk of ∼512- to 1024-cell stage embryos. For both injections, ∼1 nl containing 3.5 ng MO was used. Both *tbxta*- and control MO-injected embryos were treated with 5 µM geraniol between 8 and 24 hpf or left untreated. Ectopic tails were scored at 48 hpf.

## Supplementary Material



10.1242/develop.204791_sup1 Supplementary information
