# Supplementary material for: Migration of Kupffer's vesicle-derived cells is essential for tail morphogenesis in zebrafish embryos
Source: Development. 2025 Jun 19;152(12):dev204791. doi: 10.1242/dev.204791 (PMC12211557; doi:10.1242/dev.204791)
Supplement: Supplementary information [file develop-152-204791-s1.pdf]

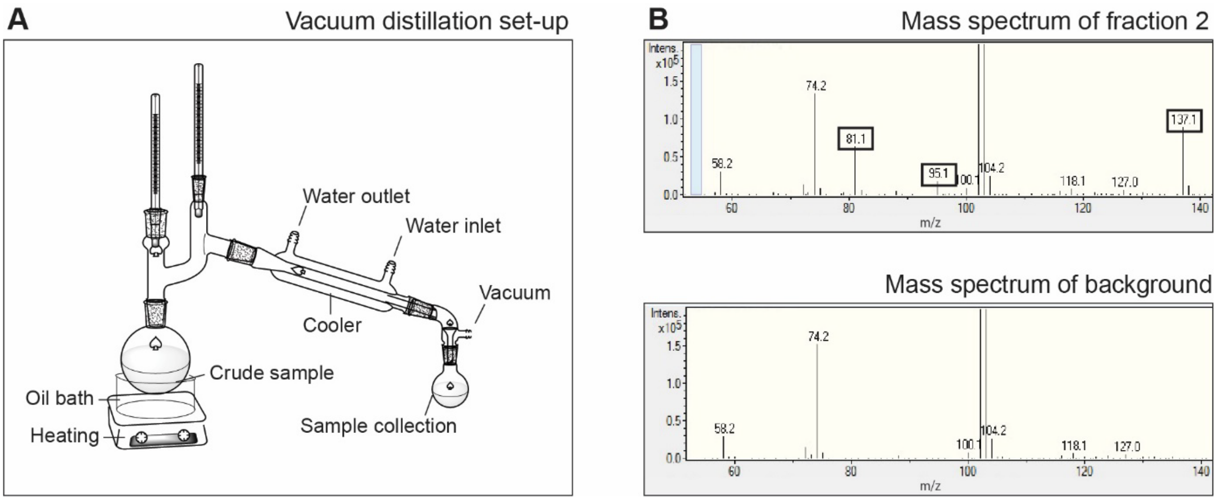

Fig. S1. Isolation and identification of ectopic tail inducing compound. (A) Representation of vacuum distillation set-up. (B) Mass spectrogram of distillation fraction 2 compared to a background spectrum reveals monoterpene alcohol specific peaks.

Development • Supplementary information

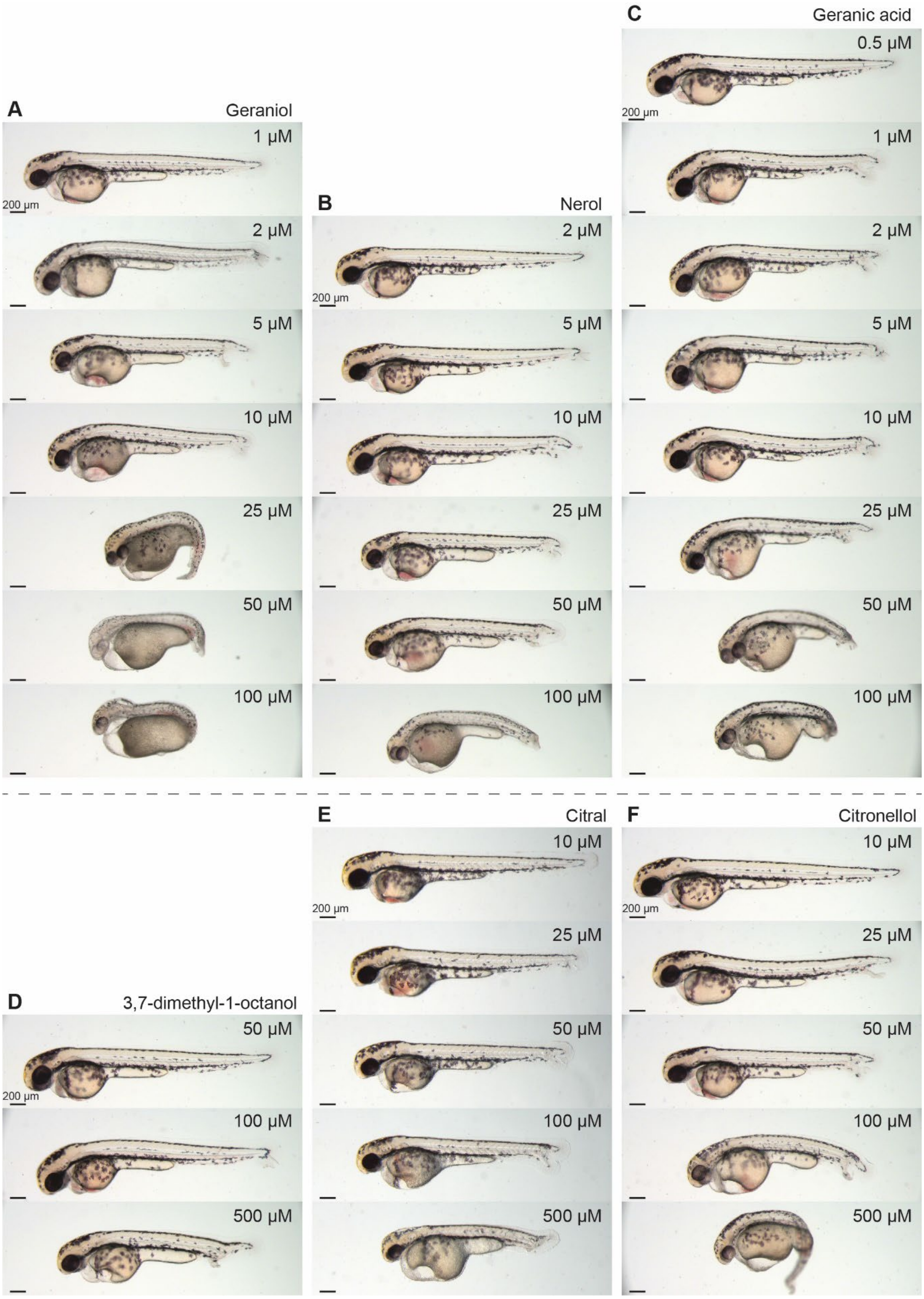

Fig. S2. Concentration-dependent ectopic tail formation by monoterpenes. Phenotypes induced by treatment of zebrafish embryos from 8 hpf onwards with increasing concentrations of monoterpenes, including (A) geraniol (B) nerol (C) geranic acid (D) 3,7-dimethyl-1-octanol (E) citral and (F) citronellol. The lowest concentration for each monoterpene shown here is the highest concentration that did not induce ectopic tail formation. Scale bar = 200 µm.

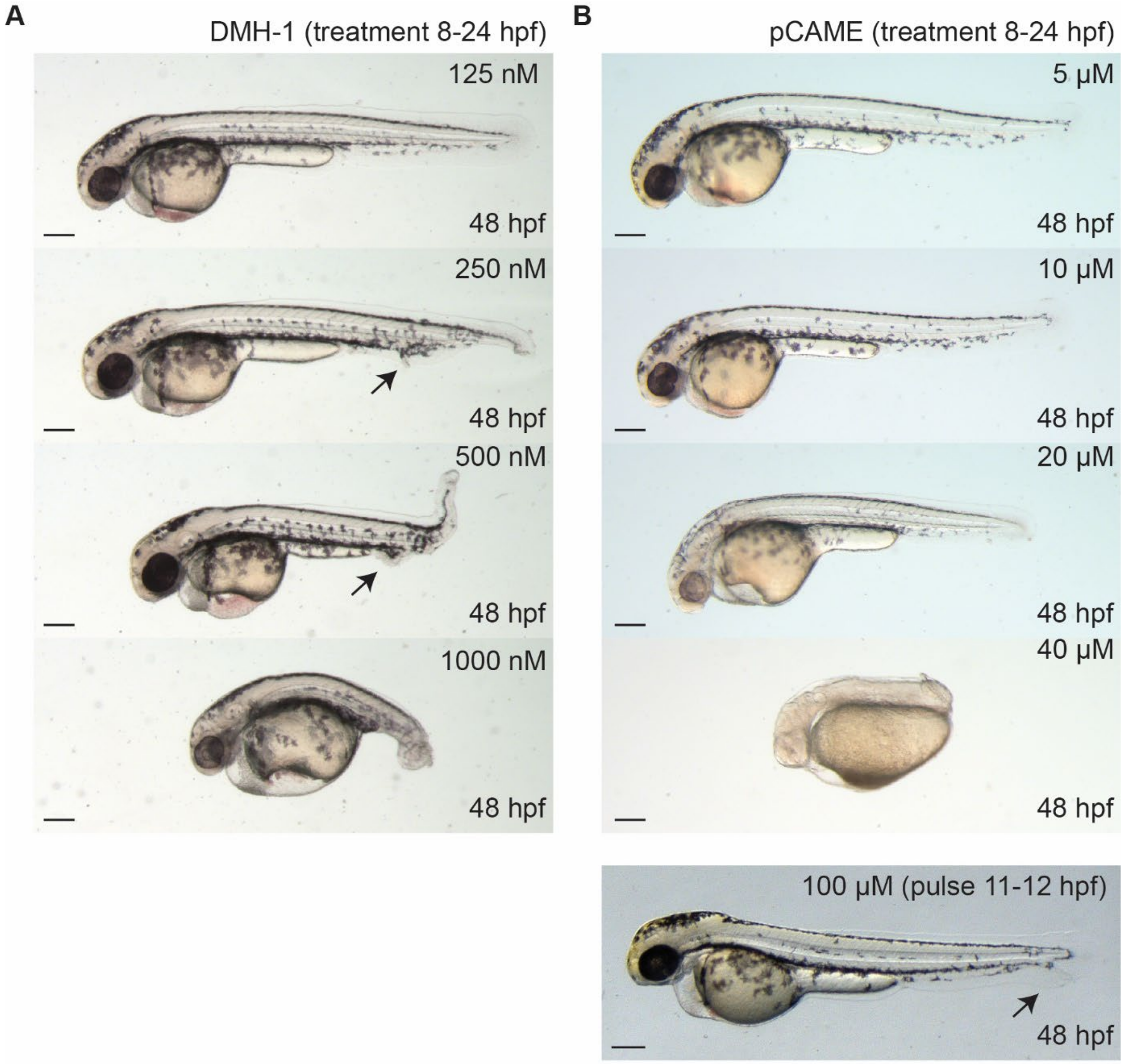

Fig. S3. Tail defects induced by DMH-1 and PCAME. (A) Phenotypes induced by increasing concentrations of BMP-inhibitor DMH-1, including loss of ventral fins and ectopic tissue formation in some embryos as indicated by the arrows. (B) Phenotypes induced by increasing concentrations of para-coumaric acid methyl ester (pCAME). pCAME only induced ectopic tails when applying 1h pulse treatments between 11-12 hpf. Scale bar = 200 μm.

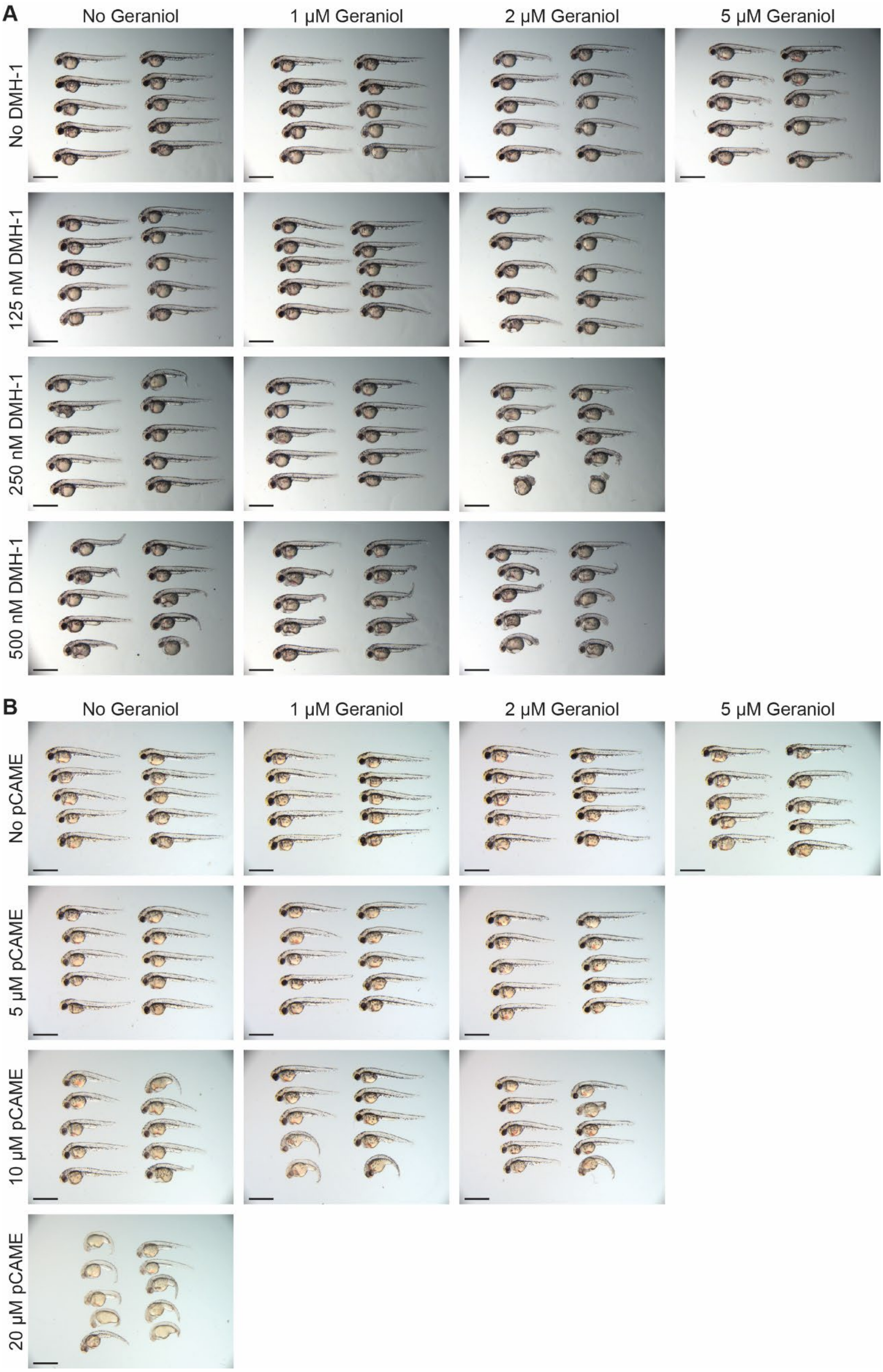

Fig. S4. Geraniol does not cooperate with DMH-1 and pCAME. (A) Combination treatments of various concentrations of geraniol and DMH-1. (B) Combination treatments of various concentrations of geraniol and pCAME. Scale bar = 200 μm.

Development • Supplementary information

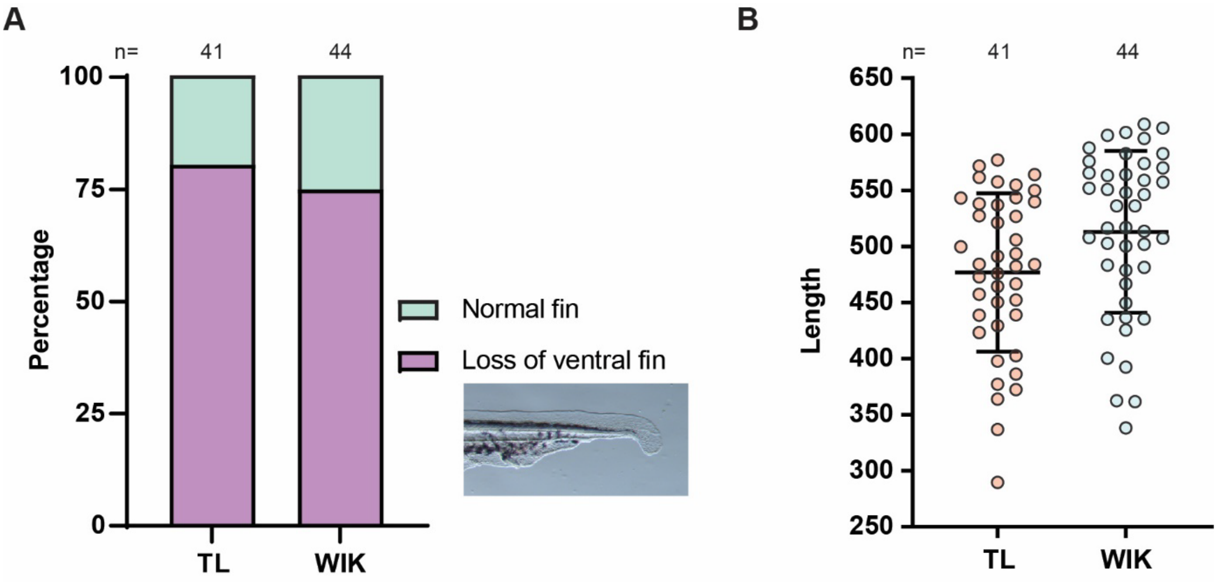

Fig. S5. DMH-1 induces a similar phenotype in TL and WIK embryos. (A) Phenotype frequency induced by DMH-1 in TL and WIK embryos. (B) Quantification of zebrafish embryo length in arbitrary units at 48 hpf per zebrafish strain in untreated and DMH-1 treated embryos.

Development • Supplementary information

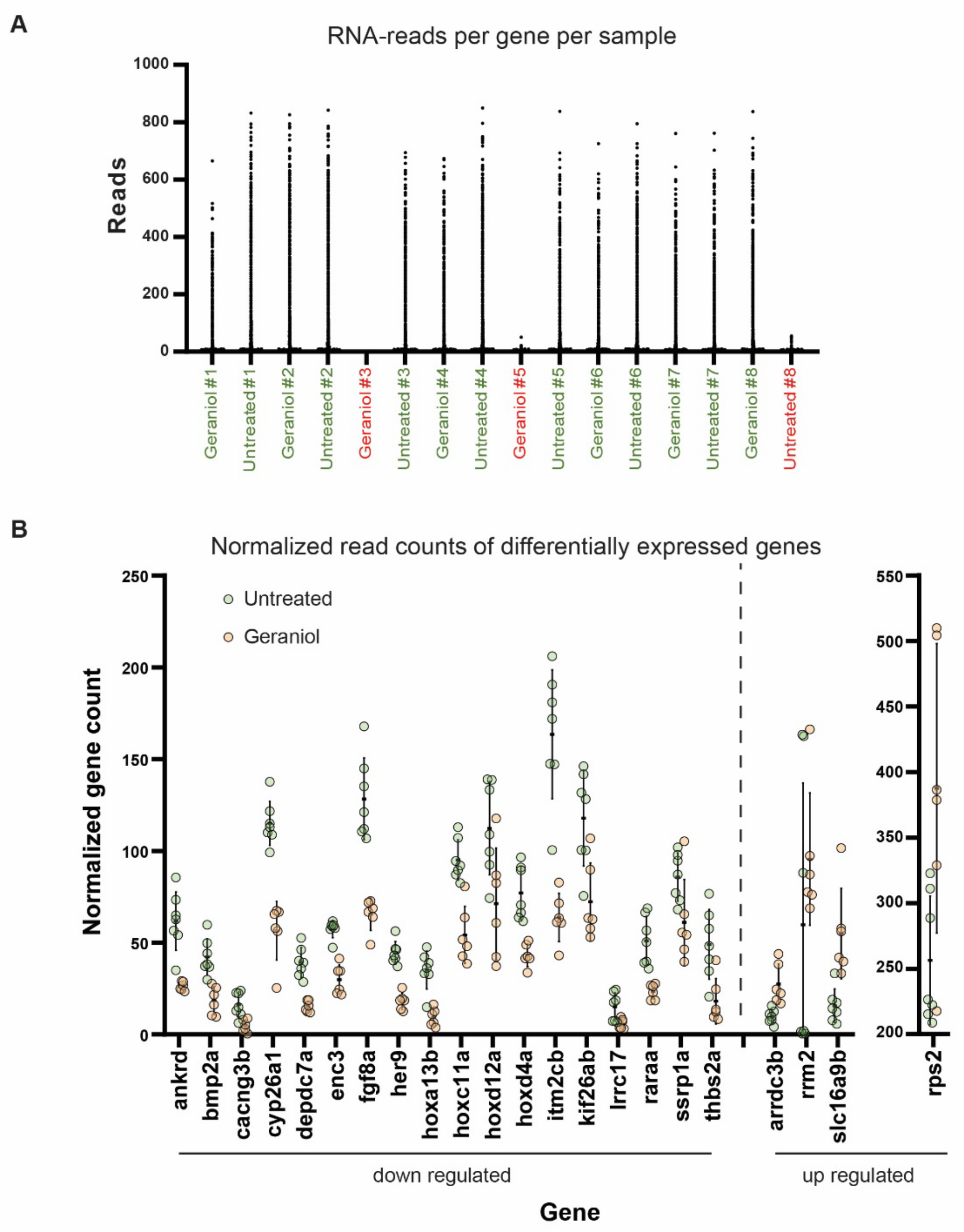

Fig. S6. RNA quality control and RNA expression counts. (A) Reads per gene per sample of the 8 control (untreated) and 8 geraniol-treated (5 µM, from 8 hpf onwards). Samples with a low number of reads (in red) were omitted from the RNA-sequencing results. (B) Normalized gene counts of differentially expressed genes.

Development • Supplementary information

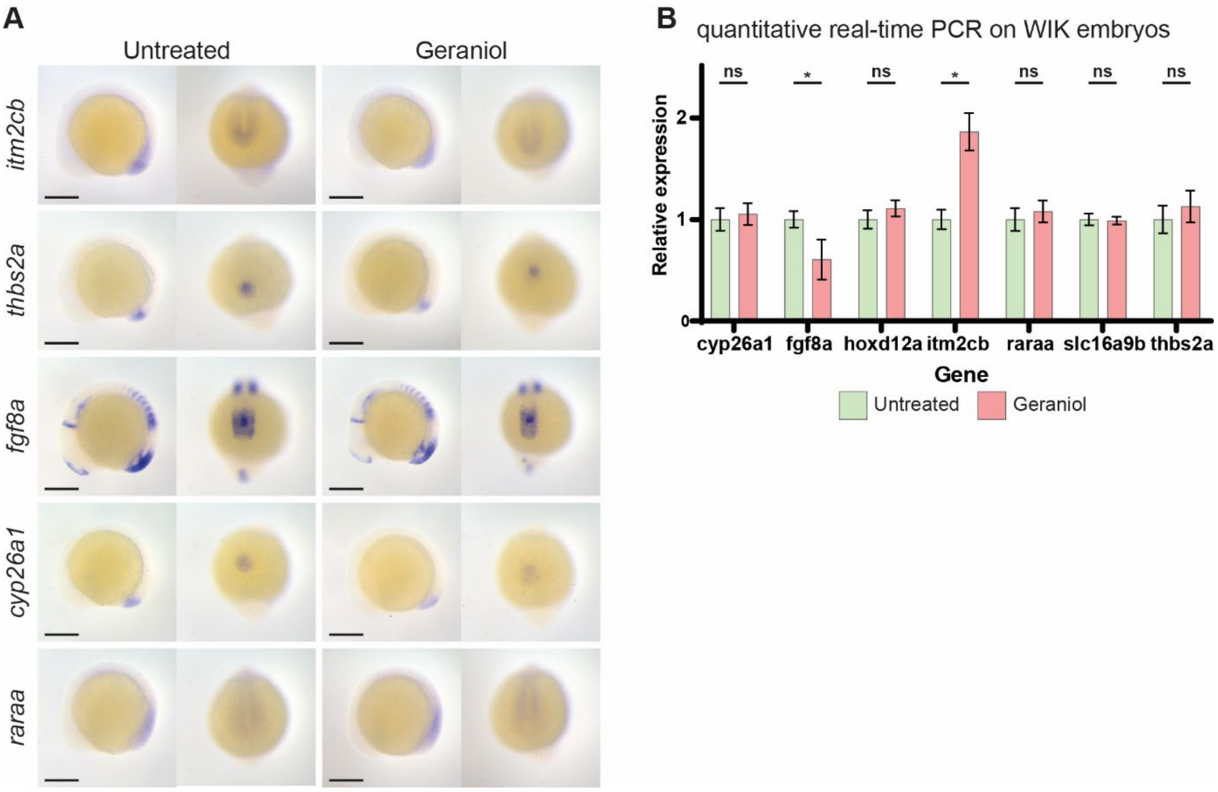

Fig. S7. Lack of differential gene expression in response to geraniol treatment in WIK embryos. (A) *In situ* hybridization using WIK embryos and probes specific for selected genes that were differentially expressed in TL embryos (Fig. 4B). Note that no clear reduction in expression was observed upon geraniol treatment. (B) Quantitative real-time PCR on 15-somite stage WIK embryos (5 embryos per sample). Only a small decrease in *fgf8a* expression and an increase in *itm2cb* expression were observed.

Development • Supplementary information

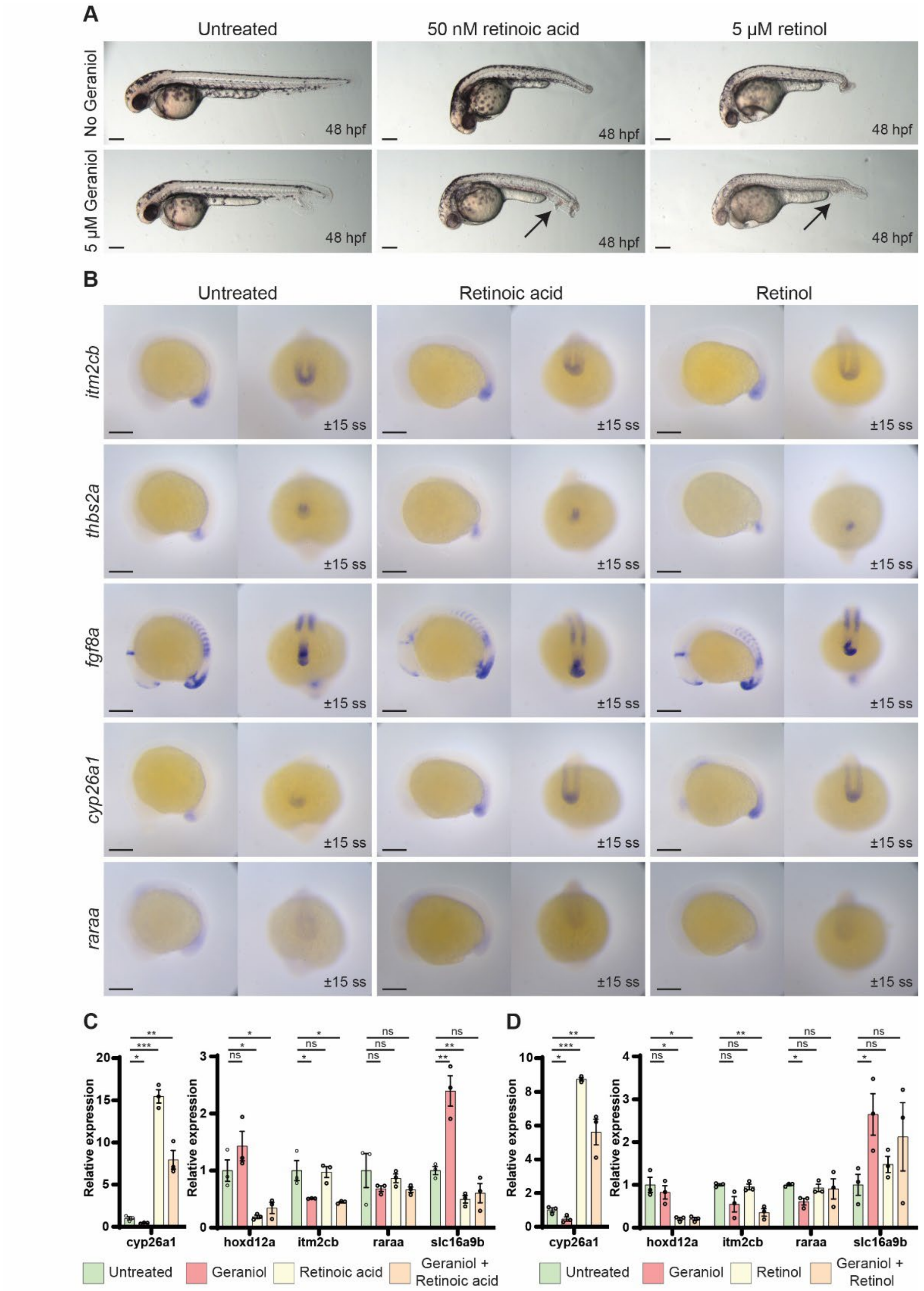

Fig. S8. Geraniol treatment does not cooperate or interfere with retinoic acid and/ or retinol treatment of embryos. (A) Tail defects induced by 50 nM retinoic acid or 5 μM retinol (8-24 hpf treatment), imaged at 48 hpf, without and with co-treatment with geraniol (5 μM). (B) *In situ* hybridization of TL embryos treated with retinoic acid or retinol using probes specific for selected genes that were differentially expressed in TL embryos in response to geraniol (Fig. 4B). A clear increase in *cyp26a1* expression was observed. Several embryos showed a skewed expression of *fgf8a* around Kupffer's vesicle. (C) Quantitative real-time PCR on whole 15-somite stage embryos (5 embryos per sample) untreated or treated with either geraniol, retinoic acid or co-treated with 5 μM geraniol and 50 nM retinoic acid. (D) Quantitative real-time PCR on whole 15-somite stage embryos (5 embryos per sample) untreated or treated with either geraniol, retinoic acid or co-treated with 5 μM geraniol and 5 μM retinol.

Development • Supplementary information

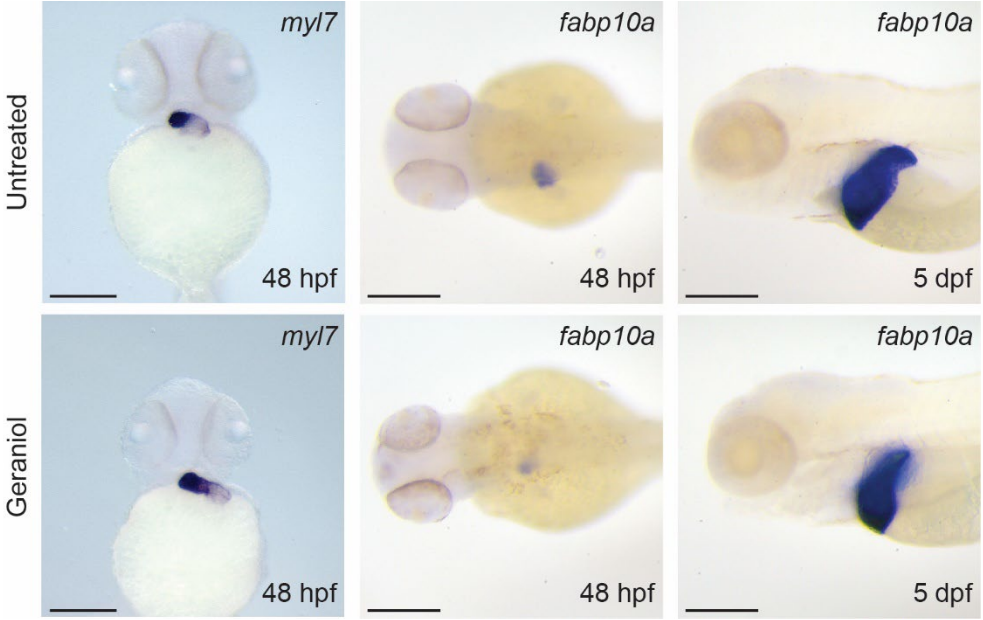

Fig. S9. Geraniol does not affect left-right asymmetry. *In situ* hybridization using TL embryos (48 hpf and 5 dpf) that had or had not been treated with geraniol (5 µM, 8-24 hpf) and probes specific for *myl7* (cardiomyocytes) or *fabp10a* (liver). It is evident that left-right asymmetry was not affected by geraniol treatment. However, liver size was reduced at 48 hpf.

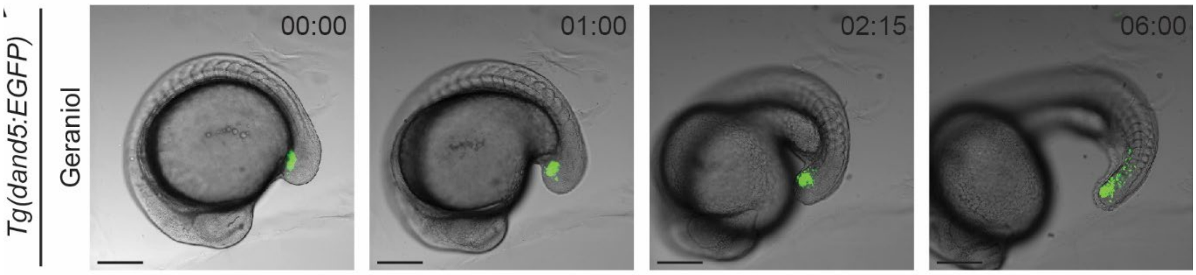

Fig. S10. Normal posterior migration of KVDCs in geraniol-treated WIK embryos. Stills from timelapse imaging using *Tg(dand5:EGFP)* embryos in WIK background, treated with geraniol (5 µM from 8 hpf onwards). 00:00 (hh:mm) corresponds to approximately 12-somite stage.

Development • Supplementary information

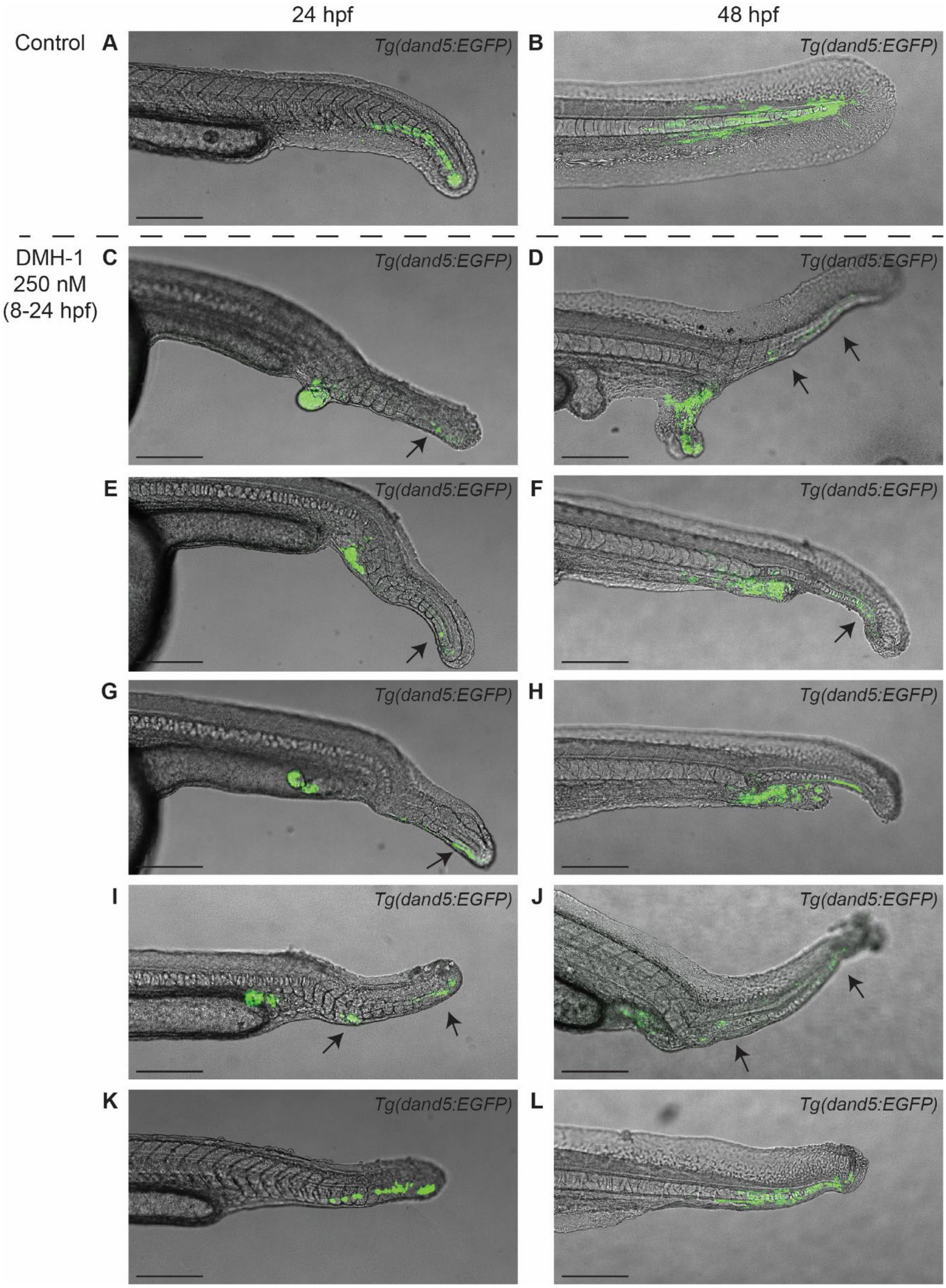

Fig. S11. KVDC distribution is variably altered in DMH-1 treated embryos. (A) Untreated *Tg*(*dand5:EGFP)* at 24 hpf. (B) Untreated *Tg*(*dand5:EGFP)* at 48 hpf. (C) *Tg*(*dand5:EGFP)* embryo treated with 250 nM DMH-1 at 24 hpf displaying an ectopic tail. (D) *Tg*(*dand5:EGFP)* embryo treated with 250 nM DMH-1 at 48 hpf displaying an ectopic tail. (E-J) *Tg*(*dand5:EGFP)* embryos treated with 250 nM DMH-1 at 24 hpf or 48 hpf with altered KVDC distribution without ectopic tail. (K,L) *Tg*(*dand5:EGFP)* embryos treated with 250 nM DMH-1 at 24 hpf or 48 hpf with regular KVDC distribution. Arrows indicate small clusters of GFP-positive cells. Scale bar = 200 μm.

Development • Supplementary information

Table S1. Chemicals & Reagents

Reagent	Supplier	Catalogue #
3,7-dimethyl-1-octanol	Merck	W239100
3,7-dimethyl-6-octenamide	Merck	S798355
Anti-Digoxigenin-AP, Fab fragments	Merck	11093274910
a-Terpineol	Merck	4899
Citral	Merck	W230308
Citronellol	Merck	W2302901
DIG RNA Labeling Mix	Merck	11277073910
Dimethyl sulfoxide	Merck	D8418
DMH-1	Merck	D8946
Dorsomorphin	Merck	P5499
Eucalyptol	Merck	C80601
FastStart Universal SYBR Green Master	Merck	4913914001
Geranic acid, 98% (sum of isomers)	Fisher	11433028
Geraniol	Merck	163333
Linalool	Merck	L2602
Methyl cellulose	Merck	M0387
MS222	Merck	E10521
Nerol	Merck	50949
p-Coumaric acid	Merck	C9008
Paraformaldehyde	Merck	158127
Retinoic acid	Merck	R2625
Retinol	Merck	R7632
TRIzol™ Reagent	Fisher	15596018

Table S2. primer sequences for probe synthesis

Gene	Forward primer sequence	Reverse primer sequence
bmp2a	AGCAGAGCCAACACTATCAG	GAG*TAATACGACTCACTATAGGG*TTAAGATGACCCGCTCGTAC
col2a	*from plasmid*	
cyp26a1	TCTACAAGACGCACCTCTTC	GAG*TAATACGACTCACTATAGGG*ACTTCTTTCATCGCCTGCAAA
fabp10a	GTGGCAGGTTTACGCTCAGG	GAG*TAATACGACTCACTATAGGG*GCTCTTCCTGATCATGGTGG
fgf8a	*from plasmid*	
hoxd12a	CCGCAGATGTCCTACAGTAG	GAG*TAATACGACTCACTATAGGG*AGTTCCAATCTGTCCGAAAGC
itm2cb	GTCTCCCTTCAGTGGCCTTT	GAG*TAATACGACTCACTATAGGG*GCGTCTCAGTCTGTAGGTCT
msgn1	CGTGAGGACAGGTCATTTGG	GAG*TAATACGACTCACTATAGGG*CGAGGATGCCGGATAACTCT
myl7	*from plasmid*	
myod1	TTCTACGACGACCCTTGCTT	GAG*TAATACGACTCACTATAGGG*TCCGTCTTCTCGTCTGACAC
ngn1	*from plasmid*	
raraa	CCTGCCTCGACATACTGATT	GAG*TAATACGACTCACTATAGGG*AGAGCACACAGTAGTCCGTAA
tbxta	*from plasmid*	
thbs2a	CCGTCAGCTTGAGATAGTGT	GAG*TAATACGACTCACTATAGGG*CATGACCTGCCTCTTTGTTG
xirp2a	CAGTACCTCAAATGCTCAGG	GAG*TAATACGACTCACTATAGGG*AATATCTCTGCTTGCCTCCTC

Table S3. Quantitative PCR primer sequences

Gene	Forward primer sequence	Reverse primer sequence
β-actin	TTCTGGTCGGTACTACTGGTATTGTG	ATCTTCATCAGGTAGTCTGTCAGGT
cyp26a1	TGTTAATGATCCGACGAGTC	CACATTATCAGCTCCCATCA
fgf8a	TACAAACGCAGGACTTTACA	TCACCTTACTTTGCTCACTC
hoxd12a	CCAGAATATCAGACCAGCTT	ACCAGATTTTGACTTGCTGA
itm2cb	AAATCCAAATGCCGTACTCT	TATCTGTAGAGGCACACTGA
raraa	GAGTTCAGAAGAGATCGTCC	ATGATGCAGTTCTTCTCTCG
slc16a9b	TCATTGCTAGTCCCATCTGC	TGGCAAGTGATGGTTACAGT
thbs2a	TTGTGGCCAAGGGATCTATC	TGTACCGACAGACACAGATT

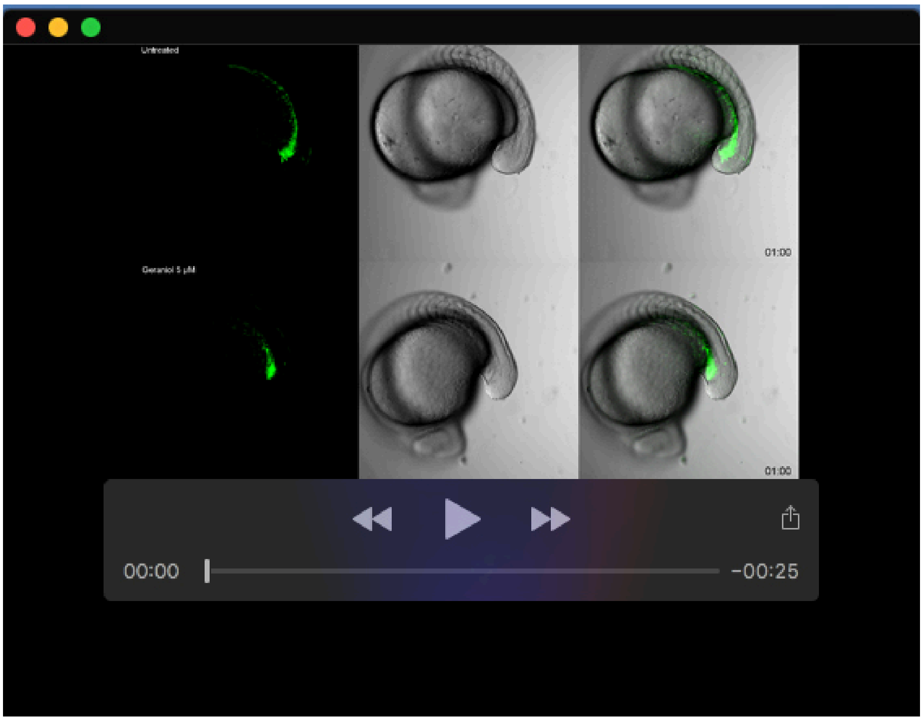

Movie 1. Geraniol inhibits Kupffer's Vesicle Derived Cell migration. *Tg(sox17:EGFP)* embryos were imaged by time lapse microscopy. Representative embryos are shown. Top: control, bottom: treated with 5 μM geraniol from 8 hpf onwards. Time is indicated (hh:mm), 00:00 represents roughly the 12-somite stage.

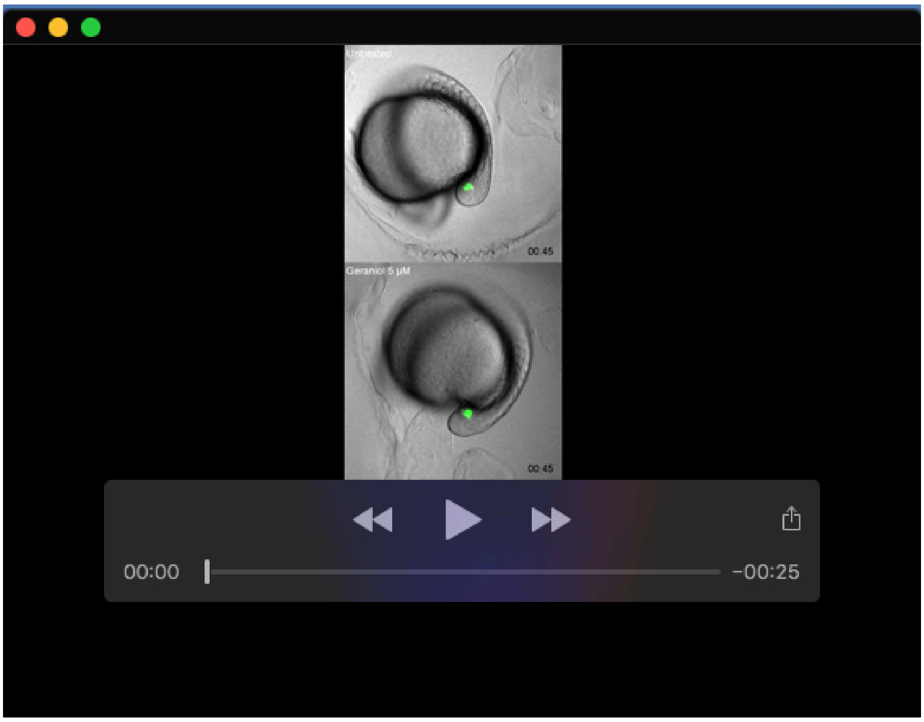

Movie 2. Geraniol inhibits Kupffer's Vesicle Derived Cell migration. *Tg(dand5:EGFP)* embryos were imaged by time lapse microscopy. Representative embryos are shown. Top: control, bottom: treated with 5 μM geraniol from 8 hpf onwards. Time is indicated (hh:mm), 00:00 represents roughly the 12-somite stage.
