# Supplementary material for: Migration of Kupffer's vesicle-derived cells is essential for tail morphogenesis in zebrafish embryos
Source: Development. 2025 Jun 19;152(12):dev204791. doi: 10.1242/dev.204791 (PMC12211557; doi:10.1242/dev.204791)
Supplement: Supplementary file 1 [file develop-152-204791_review_history.pdf]

Migration of Kupffer's vesicle-derived cells is essential for tail morphogenesis in zebrafish embryos

Jelmer Hoeksma and Jeroen den Hertog
DOI: 10.1242/dev.204791

Editor: Steve Wilson

Review timeline
Original submission: 9 July 2024
Editorial decision: 20 September 2024
Resubmission: 18 March 2025
Editorial decision: 14 May 2025
First revision received: 20 May 2025
Accepted: 23 May 2025

Original submission

Decision letter

MS ID#: dev.204237
MS TITLE: Migration of Kupffer's vesicle derived cells is essential for tail morphogenesis in zebrafish embryos

AUTHORS: Jelmer Hoeksma; Jeroen den Hertog
ARTICLE TYPE: Research Article

Dear Jerome,

I have now received referees' reports on the above manuscript, and have reached a decision. I am sorry to say that the outcome is not a positive one. The referees' comments are appended below, or you can access them online: please go to.

As you will see, Referee 1 considers that your study does not clarify how/why geraniol affects KV cells nor provides sufficient insight into the mechanistic links between KV and tail formation to justify publication in Development. Referee 2 is more positive but in cross-referee discussions, agreed with the issues raised by reviewer 1. Given these opinions, I must therefore, reject your paper.

I do realise this is disappointing news, but Development receives many more papers than we can publish, and we can only accept manuscripts that receive strong support from referees.

I do hope you find the comments of the referees helpful, and that this decision will not dissuade you from considering Development for publication of your future work. Many thanks for sending your manuscript to Development.

Reviewer 1
SUMMARY OF THE ADVANCE MADE IN THIS PAPER AND ITS POTENTIAL SIGNIFICANCE TO THE FIELD

© 2025. Published by The Company of Biologists under the terms of the Creative Commons Attribution License (https://creativecommons.org/licenses/by/4.0/).

This paper reports on the identification of geraniol as a chemical compound inducing ectopic tail formation in zebrafish. The authors show that treating embryos at mid-somite stage triggers ectopic tail formation and that this effect is not directly related to changes in retinoic acid or BMP signaling. They further show that KV disassembly and subsequent KV EMT and cell migration are defective in geraniol-treated embryos.

SUGGESTIONS TO AUTHORS

Two major weaknesses limit my enthusiasm for this work:

1. The effector mechanisms by which geraniol affects KV disassembly and KV cell migration remain unclear. Is the expression of any known regulators of EMT changed in geraniol-treated embryos? Some insight into the function of geraniol in these processes would help.

2. The role of KV disassembly and KV cell migration for tail formation (both in the wt and treated embryo) remains unknown. Thus, the functional significance of these processes for ectopic tail formation in treated embryos is unclear. The authors need to show how interference with KV EMT and/or cell migration by means other than geraniol-treatment affects tail formation. Possible experiments include ectopic induction of KV or KV ablation.

Collectively, this study does not provide sufficient insight into the activity of geraniol in early embryogenesis and/or the role of KV in tail formation.

Reviewer 2

SUMMARY OF THE ADVANCE MADE IN THIS PAPER AND ITS POTENTIAL SIGNIFICANCE TO THE FIELD

The manuscript by Hoeksma and den Hertog follows up on previous work from the lab screening for zebrafish embryonic phenotypes after treatment with fungal filtrates. Here the authors focused on an ectopic tail phenotype caused by metabolites from Ceratocystis populicola and identified monoterpene alcohols as the active compounds. Much of the subsequent analysis was done using the monoterpene alcohol geraniol. The authors performed RNA-seq on treated vs. untreated tailbud tissue and identified several genes with significantly changed expression, however this did not lead to a molecular mechanism causing the ectopic tail phenotype. The authors turned their focus to cellular mechanisms, and identified the aberrant migration of kupffer's vesicle (KV) cells in geraniol treated embryos. KV cells failed to disperse in geraniol treated embryos and ended up within the ectopic tail, suggesting that the mismigration of the KV cells is causing the ectopic tail phenotype. The manuscript presents an interesting finding, however I suggest a few more experiments to clarify the findings and to better support the conclusions.

SUGGESTIONS TO AUTHORS

Experimental suggestions:

1. It is not clear whether the KV cell mismigration is causing the ectopic tail phenotype, or alternatively that there is a change to the surrounding cells that causes the KV cells to end up in the ectopic tail. There is a straightforward method to inhibit KV formation using ntl (tbxta) morpholino injected into the yolk of mid-blastula stage embryos, targeting uptake in the dorsal forerunner cells (see Amack and Yost, 2004). If ectopic tails do not form in geraniol treated embryos that lack KV, this would show this cell population is inducing the ectopic tail.
2. To what extent do the KV cells act as a tail organizer on surrounding cells? Examining whether notochord and muscle within the ectopic tail is entirely KV derived vs recruited from surrounding cells would determine the organizer activity of KV.
3. The authors suggest that the geraniol induced ectopic tail is being generated through a distinct mechanism than BMP loss of function induced ectopic tail formation. There are some clear differences in phenotype between BMP loss of function embryos and geraniol treated embryos (such as with the ventral fin), but what happens to KV cells in BMP loss of function cells? The authors should treat KV reporter embryos with DMH-1 to determine whether KV cells also mismigrate, which would clarify whether the ectopic tail phenotype in BMP loss of function embryos is similarly related to KV mismigration.

© 2025. Published by The Company of Biologists under the terms of the Creative Commons Attribution License (https://creativecommons.org/licenses/by/4.0/).

Minor comments:

1. The smaller somites and shorter embryos observed in geraniol treated embryos suggest there might be a negative effect on Wnt signaling. Did the authors consider this and examine Wnt activity?
2. The conclusion of the attempted rescue experiment with cyp26a1 overexpression was that reduced cyp26a1 expression does not lead to ectopic tail formation. It should also be noted that cyp26a1 mutants do not have ectopic tails, and that alone is strong support that reduced cyp26a1 expression does not cause ectopic tails.
3. The discussion states that KV cells can only transdifferentiate into mesodermal lineages based on the lineages present in the ectopic tail. What happens in wild-type embryos, do KV cells ever contribute to neural tissue after migration? Also, is there endodermal hypochord tissue present in the ectopic tail?

Resubmission

MS ID#: dev.204791

MS TITLE: Migration of Kupffer's vesicle derived cells is essential for tail morphogenesis in zebrafish embryos

AUTHORS: Jelmer Hoeksma; Jeroen den Hertog

<u>Author's response to the reviewer's comments</u>

Point-by-point rebutal

We would like to thank the reviewers for their constructive reviews and their valuable suggestions that helped us substantiate our conclusions and thus improve our manuscript, as outlined in the point-by-point rebutal, below.

Reviewer #1.
1. The effector mechanisms by which geraniol affects KV disassembly and KV cell migration remain unclear. Is the expression of any known regulators of EMT changed in geraniol-treated embryos? Some insight into the function of geraniol in these processes would help.
We included a statement about arrdc3b, which is upregulated in the tail tip in response to geraniol (Fig. 4A). Lines 381-383: "For instance, arrdc3b is upregulated in response to geraniol (Fig. 4a).

ARRDC3 has recently been reported to inhibit EMT in triple knock-out breast cancer and has tumor suppressing properties (Soung et al., 2019). However, the function of arrdc3b remains to be determined definitively."

2. The role of KV disassembly and KV cell migration for tail formation (both in the wt and treated embryo) remains unknown. Thus, the functional significance of these processes for ectopic tail formation in treated embryos is unclear. The authors need to show how interference with KV EMT and/or cell migration by means other than geraniol-treatment affects tail formation. Possible experiments include ectopic induction of KV or KV ablation.

We have done KV ablation experiments by Ntl-morpholino injections as described by the Yost-lab. Ntl-morpholino injection at the 512-1024 cell stage led to a reduction in the number of dand5:eGFP-positive cells and prohibited ectopic tail formation in 30.3% of the embryos (see new Fig. 7), which is consistent with an important role for KVDCs in ectopic tail formation (lines 330-345). In addition, we have imaged DMH-1-treated Tg(dand5:eGFP) embryos and found that failure of KVDCs to disperse and migrate to the tail region correlates with ectopic tail formation (new Fig. S11, lines

© 2025. Published by The Company of Biologists under the terms of the Creative Commons Attribution License (https://creativecommons.org/licenses/by/4.0/).

292-304).

Collectively, this study does not provide sufficient insight into the activity of geraniol in early embryogenesis and/or the role of KV in tail formation.

Reviewer 2: SUMMARY OF THE ADVANCE MADE IN THIS PAPER AND ITS POTENTIAL SIGNIFICANCE TO THE FIELD

The manuscript by Hoeksma and den Hertog follows up on previous work from the lab screening for zebrafish embryonic phenotypes atier treatment with fungal filtrates. Here the authors focused on an ectopic tail phenotype caused by metabolites from Ceratocystis populicola and identified monoterpene alcohols as the active compounds. Much of the subsequent analysis was done using the monoterpene alcohol geraniol. The authors performed RNA-seq on treated vs. untreated tailbud tissue and identified several genes with significantly changed expression, however this did not lead to a molecular mechanism causing the ectopic tail phenotype. The authors turned their focus to cellular mechanisms, and identified the aberrant migration of kupffer's vesicle (KV) cells in geraniol treated embryos. KV cells failed to disperse in geraniol treated embryos and ended up within the ectopic tail, suggesting that the mismigration of the KV cells is causing the ectopic tail phenotype. The manuscript presents an interesting finding, however I suggest a few more experiments to clarify the findings and to beter support the conclusions.

SUGGESTIONS TO AUTHORS

Experimental suggestions:

1. It is not clear whether the KV cell mismigration is causing the ectopic tail phenotype, or alternatively that there is a change to the surrounding cells that causes the KV cells to end up in the ectopic tail. There is a straightiorward method to inhibit KV formation using ntl (tbxta) morpholino injected into the yolk of mid-blastula stage embryos, targeting uptake in the dorsal forerunner cells (see Amack and Yost, 2004). If ectopic tails do not form in geraniol treated embryos that lack KV, this would show this cell population is inducing the ectopic tail.

We have performed the experiments suggested by the reviewer and found that embryos that lack KV do not form an ectopic tail in response to geraniol treatment (new Fig. 7, lines 330-345).

2. To what extent do the KV cells act as a tail organizer on surrounding cells? Examining whether notochord and muscle within the ectopic tail is entirely KV derived vs recruited from surrounding cells would determine the organizer activity of KV.

We have done double labeling of Tg(dand5:GFP) embryos by immunohistochemistry of GFP and in situ hybridization using three distinct cell fate-specific probes, showing co-staining of the cells, which provides evidence that KVDCs contribute to different cell fates (new Fig. 6C; lines 319-323). We cannot exclude the possibility that KVDCs also recruit surrounding cells to form the ectopic tail. It appears that not all cells in the ectopic tail are KVDC-derived.

3. The authors suggest that the geraniol induced ectopic tail is being generated through a distinct mechanism than BMP loss of function induced ectopic tail formation. There are some clear differences in phenotype between BMP loss of function embryos and geraniol treated embryos (such as with the ventral fin), but what happens to KV cells in BMP loss of function cells? The authors should treat KV reporter embryos with DMH-1 to determine whether KV cells also mismigrate, which would clarify whether the ectopic tail phenotype in BMP loss of function embryos is similarly related to KV mismigration.

We have imaged KVDCs in tg(dand5:eGFP) embryos treated with DMH-1 (Fig. S11). The data indicate that KVDCs mismigrate in response to DMH-1 and depending on the location of the KVDCs, the embryos generate an ectopic tail. The migration patern is different than in response to geraniol, bolstering the conclusion that the response to geraniol is distinct from DMH-1-mediated inhibitionof BMP signaling (lines 292-304).

© 2025. Published by The Company of Biologists under the terms of the Creative Commons Attribution License (https://creativecommons.org/licenses/by/4.0/).

Minor comments:

1. The smaller somites and shorter embryos observed in geraniol treated embryos suggest there might be a negative effect on Wnt signaling. Did the authors consider this and examine Wnt activity?

We did consider Wnt signaling. However, Wnt expression was very low in the RNAseq data and in situ hybridization with a wnt3a-specific probe did not lead to promising results. Therefore, we have not pursued this interesting option further.

2. The conclusion of the atempted rescue experiment with cyp26a1 overexpression was that reduced cyp26a1 expression does not lead to ectopic tail formation. It should also be noted that cyp26a1 mutants do not have ectopic tails, and that alone is strong support that reduced cyp26a1 expression does not cause ectopic tails.

Indeed, the lack of ectopic tails in cyp26a mutants is a strong indication that the lack of Cyp26a by itself does not induce ectopic tails. In addition, we verified that restoration of reduced expression of cyp26a does not restore ectopic tail formation, indicating that it is unlikely that loss of cyp26a expression is involved in ectopic tail formation.

3. The discussion states that KV cells can only transdifferentiate into mesodermal lineages based on the lineages present in the ectopic tail. What happens in wild-type embryos, do KV cells ever contribute to neural tissue atier migration? Also, is there endodermal hypochord tissue present inthe ectopic tail?

According to Ikeda et al. , dand5:EGFP positive cells do not contribute to the neural tube, but do contribute to the notochord. The data we have collected are consistent with this observation. Col2a, a marker of the hypochord is expressed in the ectopic tail, but ngn1, which marks neural ectoderm is not expressed in ectopic tails (Fig. 6B, lines 307-318).

First decision letter

MS ID#: dev.204791

MS TITLE: Migration of Kupffer's vesicle derived cells is essential for tail morphogenesis in zebrafish embryos

AUTHORS: Jelmer Hoeksma; Jeroen den Hertog

Dear Jeroen,

I have now received all the referees reports on the above manuscript, and have reached a decision. Please note that one of the original referees was not able to review this version and so I asked a new reviewer for their opinion. The referees' comments are appended below, or you can access them online: please go to.

The overall evaluation is positive and we would like to publish a revised manuscript in Development, provided that the referees' comments can be satisfactorily addressed. Please attend to all of the reviewers' comments in your revised manuscript and detail them in your point-by-point response. If you do not agree with any of their criticisms or suggestions explain clearly why this is so. If it would be helpful, you are welcome to contact us to discuss your revision in greater detail. Please send us a point-by-point response indicating your plans for addressing the referees' comments, and we will look over this and provide further guidance.

Reviewer 1

© 2025. Published by The Company of Biologists under the terms of the Creative Commons Attribution License (https://creativecommons.org/licenses/by/4.0/).

The original reviewers highlighted that this is potentially exciting work. They raised concerns regarding how much insight the study provides about tail formation. To address this, the authors have added new data, particularly using ntl morpholino. This data is potentially informative, but it is not presented in a sufficiently clear manner (see comments below).

Comments

1. Figure 3A. The variability between starting the incubation at 13 or 14 hpf (13 is orange and 14 is green) is likely due to low experiment number (5 per sample). Rather than a simple color coding, it would be better to show the actual fraction measured (1/5, 2/5 etc.).
2. Figure 4B - highlighting specific differences would help (e.g. ,with arrows) rather than relying on the reader to identify differences.
3. Figure 6C - the colors are not a good choice, making it hard to see details against the black background.
4. Figure 7A - lacks scale bars and clear identification of the green marker. This Figure needs further work. For example, the experimental data shown on the left is hard to parse with the data shown on the right. At the moment, the data presentation is insufficient to make strong conclusions (for example, the claim "observed a variation of phenotypes at the 15-somite stage ranging from embryos with a reduced number of disorganized dand5:EGFP positive cells to apparently normal Kupffer's vesicle development (Fig. 7A)." is not supported by the data shown in Figure 7A.

Clear examples should be shown of the different phenotypes, especially for the Ger ntl treatment. It is necessary to show clear images of the rescue embryos in the ntl-MO- geraniol treated conditions.

Reviewer 2

SUMMARY OF THE ADVANCE MADE IN THIS PAPER AND ITS POTENTIAL SIGNIFICANCE TO THE FIELD

The authors have addressed my major experimental recommendations from the 1st submission. Most notably, they assayed ectopic tail formation in geraniol treated embryos when kupfurr's vesicle (KV) cells are ablated using the tbxta morpholino. The loss of ectopic tails in this condition indicates the importance of KV cell mis-migration in causing the ectopic tail phenotype, and suggests that KV cells have the capacity to form an ectopic tail (minus the neural tissue). The analysis of KV cells in BMP inhibitor treated embryos also shows that ectopic tails in this manipulation is also likely the result of KV mis-migration. Although the BMP inhibitor and geraniol treated embryos have distinct phenotypes suggesting geraniol is not inhibiting BMP signaling, the common presence of mis-migrated KV cells in ectopic tails in both conditions further illustrates the importance of proper KV migration. The manuscript has improved considerably based on these new additions.

SUGGESTIONS TO AUTHORS

Minor comment: since ntl is currently referred to as tbxta, it would be good to either refer to it as this or indicate at the beginning that another name for ntl is tbxta.

First revision

Author response to reviewers' comments

Reviewer 1:

1. Figure 3A. The variability between starting the incubation at 13 or 14 hpf (13 is orange and 14 is green) is likely due to low experiment number (5 per sample). Rather than a simple color coding, it would be better to show the actual fraction measured (1/5, 2/5 etc.).

© 2025. Published by The Company of Biologists under the terms of the Creative Commons Attribution License (https://creativecommons.org/licenses/by/4.0/).

Next to the bars in the figure, we have indicated the actual number of embryos that developed an ectopic tail of the total number of embryos.

2. Figure 4B - highlighting specific differences would help (e.g. ,with arrows) rather than relying on the reader to identify differences.

We have highlighted the differences with arrows.

3. Figure 6C - the colors are not a good choice, making it hard to see details against the black background.

Some of the signals are rather weak. We have changed red to magenta to increase the contrast.

4. Figure 7A - lacks scale bars and clear identification of the green marker. This Figure needs further work. For example, the experimental data shown on the left is hard to parse with the data shown on the right. At the moment, the data presentation is insufficient to make strong conclusions (for example, the claim "observed a variation of phenotypes at the 15-somite stage ranging from embryos with a reduced number of disorganized dand5:EGFP positive cells to apparently normal Kupffer's vesicle development (Fig. 7A)." is not supported by the data shown in Figure 7A.

Clear examples should be shown of the different phenotypes, especially for the Ger ntl treatment. It is necessary to show clear images of the rescue embryos in the ntl-MO- geraniol treated conditions.

We have included scale bars in panel A. We have included examples of the phenotypes in the new panel B and indicated the frequencies of these phenotypes.

Reviewer 2: SUMMARY OF THE ADVANCE MADE IN THIS PAPER AND ITS POTENTIAL SIGNIFICANCE TO THE FIELD

SUGGESTIONS TO AUTHORS

Minor comment: since ntl is currently referred to as tbxta, it would be good to either refer to it as this or indicate at the beginning that another name for ntl is tbxta.

We have replaced ntl with tbxta throughout the text.

Second decision letter

MS ID#: dev.204791R1

MS TITLE: Migration of Kupffer’s vesicle derived cells is essential for tail morphogenesis in zebrafish embryos

AUTHORS: Jelmer Hoeksma; Jeroen den Hertog
ARTICLE TYPE: Research Article

Dear Jeroen,

I am happy to tell you that your manuscript has been accepted for publication in Development, pending our standard publication integrity checks.

© 2025. Published by The Company of Biologists under the terms of the Creative Commons Attribution License (https://creativecommons.org/licenses/by/4.0/).
